# Engineered extracellular vesicles for targeted reprogramming of cancer-associated fibroblasts to potentiate therapy of pancreatic cancer

**DOI:** 10.1038/s41392-024-01872-7

**Published:** 2024-06-24

**Authors:** Pengcheng Zhou, Xuanlong Du, Weilu Jia, Kun Feng, Yewei Zhang

**Affiliations:** 1 Department of General Surgery, Affiliated Hospital of Nantong University, Nantong, China; 2 School of Medicine, Southeast University, Nanjing, China; 3 Nanjing Medical University, Nanjing, China; 4 Hepatobiliary and Pancreatic Center, The Second Affiliated Hospital of Nanjing Medical University, Nanjing, China

**Keywords:** Nanobiotechnology, Cancer microenvironment, Drug delivery

## Abstract

Pancreatic cancer is one of the deadly malignancies with a significant mortality rate and there are currently few therapeutic options for it. The tumor microenvironment (TME) in pancreatic cancer, distinguished by fibrosis and the existence of cancer-associated fibroblasts (CAFs), exerts a pivotal influence on both tumor advancement and resistance to therapy. Recent advancements in the field of engineered extracellular vesicles (EVs) offer novel avenues for targeted therapy in pancreatic cancer. This study aimed to develop engineered EVs for the targeted reprogramming of CAFs and modulating the TME in pancreatic cancer. EVs obtained from bone marrow mesenchymal stem cells (BMSCs) were loaded with miR-138-5p and the anti-fibrotic agent pirfenidone (PFD) and subjected to surface modification with integrin α5-targeting peptides (named IEVs-PFD/138) to reprogram CAFs and suppress their pro-tumorigenic effects. Integrin α5-targeting peptide modification enhanced the CAF-targeting ability of EVs. miR-138-5p directly inhibited the formation of the FERMT2-TGFBR1 complex, inhibiting TGF-β signaling pathway activation. In addition, miR-138-5p inhibited proline-mediated collagen synthesis by directly targeting the FERMT2-PYCR1 complex. The combination of miR-138-5p and PFD in EVs synergistically promoted CAF reprogramming and suppressed the pro-cancer effects of CAFs. Preclinical experiments using the orthotopic stroma-rich and patient-derived xenograft mouse models yielded promising results. In particular, IEVs-PFD/138 effectively reprogrammed CAFs and remodeled TME, which resulted in decreased tumor pressure, enhanced gemcitabine perfusion, tumor hypoxia amelioration, and greater sensitivity of cancer cells to chemotherapy. Thus, the strategy developed in this study can improve chemotherapy outcomes. Utilizing IEVs-PFD/138 as a targeted therapeutic agent to modulate CAFs and the TME represents a promising therapeutic approach for pancreatic cancer.

## Introduction

Pancreatic cancer is a highly aggressive and lethal malignancy that continues to pose a significant global health challenge. It ranked as the third-highest contributor to cancer-related mortality among all cancer types.^1^ Tumor growth and treatment resistance are both influenced by the intricate relationship between cancer cells and the tumor microenvironment (TME). In the TME, cancer-associated fibroblasts (CAFs) regulate the processes involved in pancreatic cancer pathogenesis, including tumor growth, immune evasion, therapy resistance, and extracellular matrix (ECM) remodeling.^2^ Consequently, the targeted modulation of CAFs is a potential strategy to boost the efficacy of pancreatic cancer therapy.

Recently, the roles of extracellular vesicles (EVs) in intercellular communication and their potential therapeutic applications have piqued the interest of the scientific community.^3^ EVs, which are membrane-bound cellular vesicles released by cells, can deliver bioactive molecules to recipient cells. EVs can be engineered to carry therapeutic agents, such as genetic materials or drugs to the target sites through ectogenic loading. In addition, EVs can be modified to improve their cargo delivery efficiency and target specificity, which will enable the optimization of therapeutic outcomes in patients. Bone marrow mesenchymal stem cells (BMSCs), a population of stem cells with multipotency, are reported to exhibit anti-tumor^4^ and anti-fibrotic properties.^5^ The preclinical and clinical effects of BMSCs have been widely studied. The advanced extraction techniques of BMSCs have contributed to their widespread clinical acceptance. As BMSCs are autologous stem cells, they are associated with a high safety profile and minimal immune rejection. Furthermore, BMSCs are one of the potential sources of CAFs.^6^ During tumor development, BMSCs can be recruited to the TME and differentiate into CAFs.^7^ Due to their homing ability, BMSC-derived EVs (BMSC-EVs) possess both the inherent advantages of EVs and the characteristics associated with BMSCs, making them a highly promising therapeutic approach for selectively targeting CAFs in pancreatic cancer. The manipulation of cargo composition in combination with the improvement of the targeting ability of EVs will enable the specific delivery of therapeutics to CAFs, modulating their function and inhibiting their tumor-promoting effects. This strategy is a unique approach to disrupting the reciprocal crosstalk between CAFs and cancer cells, reversing the fibrotic TME, and consequently improving treatment outcomes.

MicroRNAs (miRNAs), a class of small non-coding RNA molecules, are involved in the regulation of disease development by binding to the 3’-untranslated region (3’-UTR) of mRNA, inhibiting gene translation or downregulating the target gene levels. Existing literature has demonstrated that miR-138-5p can impede cancer progression by utilizing a variety of mechanisms to suppress cancer cells.^8,9^ In addition, miR-138-5p is capable of inhibiting ZEB2 expression, reversing the fibrotic phenotype of pulmonary epithelial cells.^10^ miR-138-5p in mesenchymal stem cell (MSC)-derived EVs can target *SIRT1*, suppressing the proliferation and migration of human dermal fibroblasts and downregulating the levels of NF-κB, α-SMA, and TGF-β1.^11^ Nevertheless, there has been no previous investigation into the function of miR-138-5p in CAFs. An anti-fibrotic drug known as pirfenidone (PFD) is prescribed for the management of idiopathic pulmonary fibrosis (IPF).^12,13^ PFD possesses anti-inflammatory, anti-fibrotic, and antioxidant functions in different animal models. In addition, in the context of tumor therapy, PFD is proven to be effective in suppressing TGF-β expression, thereby suppressing the activation of CAFs and the production of collagen, consequently impeding tumor progression.^14,15^

In this study, miR-138-5p expression and function in pancreatic CAFs were investigated. Next, BMSC-EVs were loaded with PFD and miR-138-5p using ultrasonication and subjected to surface modification with integrin α5-targeting peptides to obtain engineered integrin α5-targeted EVs (IEVs) loaded with PFD and miR-138-5p (named IEVs-PFD/138). The functional mechanism of miR-138-5p was examined using the dual-luciferase reporter and co-immunoprecipitation (co-IP) assays. The impacts of IEVs-PFD/138 on the targeted reprogramming of pancreatic CAFs were examined using two-dimensional (2D) cell functional experiments, conditional medium (CM) co-culture experiments, three-dimensional (3D) multicellular tumor spheroid experiments, and subcutaneous xenograft models. Finally, the therapeutic effect of the combination of IEVs-PFD/138 and gemcitabine (GEM) was examined using the patient-derived xenograft (PDX) and orthotopic stroma-rich pancreatic cancer mouse models.

## Results

### miR-138-5p exhibits downregulated expression in pancreatic CAFs and can reprogram the phenotype of pancreatic CAFs

**Fig. 1:**
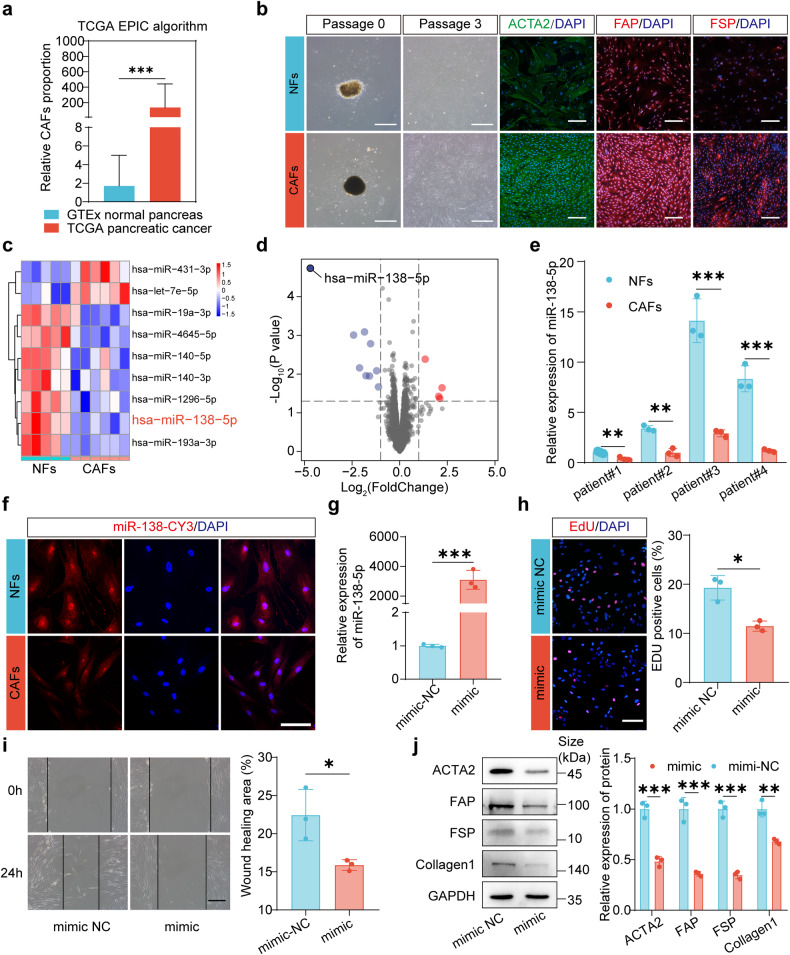
Effect of miR-138-5p on pancreatic cancer-associated fibroblasts (CAFs). **a** Analysis of CAF proportion in The Cancer Genome Atlas (TCGA) pancreatic cancer dataset and Genotype-Tissue Expression (GTEx) healthy pancreas dataset using the EPIC algorithm. **b** Morphology of CAFs and healthy fibroblasts (NFs) and immunofluorescence analysis of ACTA2, FAP, and FSP (scale bar = 100 μm). **c** Identification of differentially expressed microRNAs (miRNAs) between CAFs and NFs using the miRNA array. **d** miR-138-5p had the highest significant difference in expression between CAFs and NFs. **e** Quantitative real-time polymerase chain reaction (qRT-PCR) validation of miR-138-5p downregulation in CAFs relative to paired NFs. Data are presented as mean (± SD); *n* = 3 per group. **f** miR-138 expression in CAFs and NFs as determined by Fluorescent in situ hybridization (FISH) analysis (scale bar = 100 μm). **g** qRT-PCR validation of miR-138-5p mimic transfection efficiency. Data are presented as mean (± SD); *n* = 3 per group. **h** EdU assay of CAFs transfected with miR-138-5p mimic (scale bar = 100 μm). Data indicate the mean (± SD); *n* = 3 per group. **i** The wound-healing assay results of CAFs transfected with miR-138-5p mimic (scale bar = 100 μm). Data are presented as mean (± SD); *n* = 3 per group. **j** Western blotting analysis of the impact of miR-138-5p mimic transfection on the ACTA2, FAP, FSP, and collagen1 protein expression levels (scale bar = 100 μm). Data represent the mean (± SD); *n* = 3 per group

Analysis using the EPIC algorithm revealed that the CAF proportion in The Cancer Genome Atlas (TCGA) pancreatic cancer dataset was increased relative to that in the Gene-Tissue Expression (GTEx) healthy pancreas dataset (Fig. 1a). Primary CAFs and healthy fibroblasts (NFs) were cultured using the outgrowth method. CAFs and NFs exhibited a spindle shape. Immunofluorescence showed that CAFs had a stronger expression of ACTA2, FAP, and FSP than NFs (Fig. 1b, Supplementary Fig. 1a). The differentially expressed miRNAs were identified using the miRNA array of pancreatic CAFs and NFs (Fig. 1c). Among the differentially expressed miRNAs, miR-138-5p was found to have a great significant difference in expression between CAFs and NFs (Fig. 1d). Quantitative real-time polymerase chain reaction (qRT-PCR) proved that miR-138-5p in CAFs was downregulated when compared with that in paired NFs (Fig. 1e). Fluorescence in situ hybridization (FISH) analysis further illustrated that miR-138 expression in CAFs was lower than that in NFs (Fig. 1f). To facilitate subsequent in vitro and in vivo studies, an immortalized CAF cell line was established (Supplementary Fig. 1b–c). The function of miR-138-5p was investigated. Notably, the results demonstrated that miR-138-5p mimic transfection markedly upregulated miR-138-5p expression (Fig. 1g). In addition, the 5-bromo-2’-deoxyuridine (EdU) and CCK-8 assays demonstrated that miR-138-5p mimic decreased the proliferative rate of CAFs (Fig. 1h, Supplementary Fig. 1d). The findings from the wound-healing and transwell assays indicated that the migratory rate of CAFs was reduced by miR-138-5p mimic (Fig. 1i, Supplementary Fig. 1e). Further, the ACTA2, FAP, FSP, and collagen1 protein expression was downregulated by transfection with miR-138-5p, as determined by Western blotting analysis (Fig. 1j).

### Preparation and characterization of IEVs-PFD/138

**Fig. 2:**
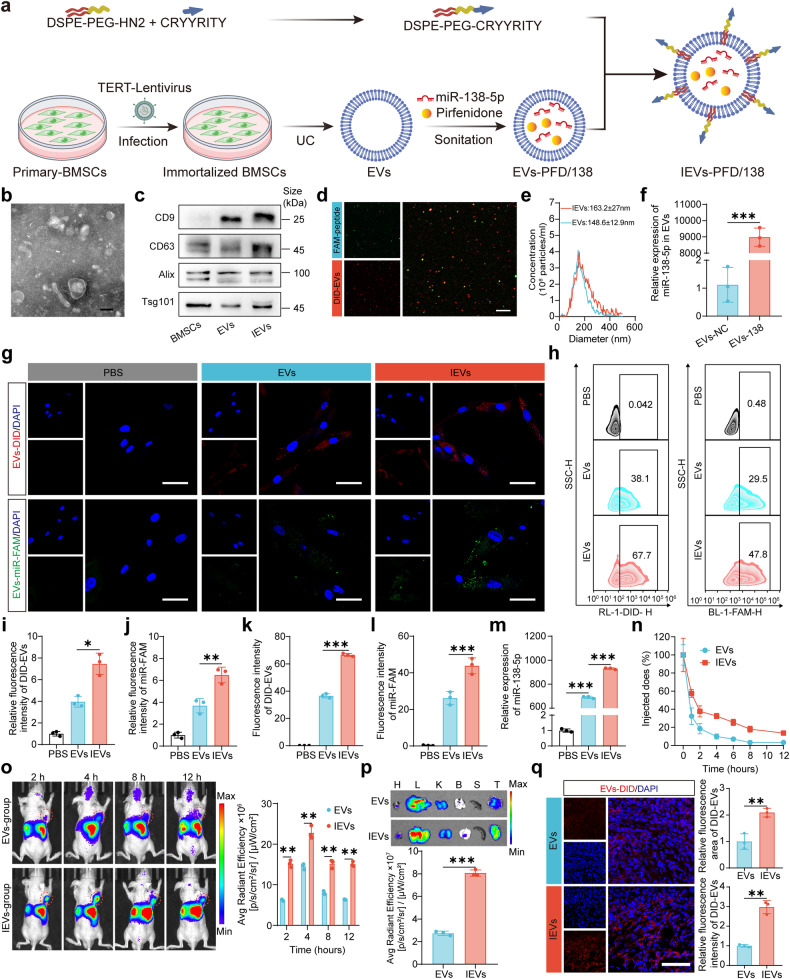
Preparation and characterization of extracellular vesicles (EVs) loaded with miR-138-5p and pirfenidone (PFD) and subjected to surface modification with integrin α5-targeting peptides (IEVs-PFD/138) and their enhanced cancer-associated fibroblast (CAF)-targeting ability. **a** Schematic representation of engineered bone marrow mesenchymal stem cell (BMSC)-derived EVs for the co-delivery of miR-138-5p mimic and PFD to pancreatic CAFs. **b** Transmission electron microscopy images of EVs (scale bar = 100 nm). **c** The EV markers (CD9, CD63, Alix, and Tsg101) in both EVs and IEVs were analyzed by Western blotting. **d** The fluorescence co-localization staining of FAM-peptide (green) and DID-labeled EVs (red). **e** Nanoparticle tracking analysis (NTA) of EVs and IEVs. **f** Quantitative real-time polymerase chain reaction (qRT-PCR) of miR-138-5p expression in EVs. Data represent the mean (±SD); *n* = 3 per group. **g**, **h** Cellular uptake of DID-labeled EVs and fluorescein amidite (FAM)-miR-138-5p-loaded EVs in CAFs. Laser scanning confocal microscopy (LSCM) (scale bar = 100 μm) and flow cytometric analyses of the uptake of IEVs by CAFs. **i**–**l** Quantification of LSCM and flow cytometric analysis results. Data are presented as mean (±SD); *n* = 3 per group. **m** qRT-PCR analysis of miR-138-5p expression in CAFs. Data represent the mean (±SD); *n* = 3 per group. **n** In vivo circulation time of IEVs and undecorated EVs. **o** Analysis of the pancreatic cancer model with in vivo Imaging System (IVIS). Data are presented as mean (±SD); *n* = 3 per group. **p** Analysis of the main organs and tumor with in vivo Imaging System (IVIS). Data are presented as mean (±SD); *n* = 3 per group. **q** Analysis of the intra-tumoral distribution of IEVs in the tumor section (scale bar = 100 μm). Data are presented as mean (±SD); *n* = 3 per group

We constructed engineered BMSCs-derived EVs for pancreatic CAFs-targeted co-delivery of the gene-drug miR-138-5p mimic and anti-fibrosis drug pirfenidone (PFD) (Fig. 2a). The primary BMSCs were infected with human telomerase reverse transcriptase (hTERT)-encoding lentivirus to construct immortalized BMSCs. The immortalized BMSCs exhibited a spindle-shaped arrangement of radial concentric circles with broom-like growth (Supplementary Fig. 2a). Immortalized BMSCs were identified based on osteogenic, adipogenic, and chondrogenic induction (Supplementary Fig. 2a). Flow cytometry showed that immortalized BMSCs negatively expressed CD45, CD14, CD11B, CD34, and HLA, but positively expressed CD29 and CD73 (Supplementary Fig. 2b). Ultracentrifugation was used to separate the EVs, and Western blotting, transmission electron microscopy (TEM), and nanoparticle tracking analysis were used to characterize them. A characteristic round morphology was observed in EVs, as shown by TEM (Fig. 2b). In addition, EVs and IEVs were positive for CD9, CD63, Alix, and Tsg101, as determined by the Western blotting analysis (Fig. 2c). To confirm the EVs were loaded with integrin α5 peptide, we labeled DSPE-PEG-CRYYRITY with FAM fluorescence and EVs with DID fluorescence. Under confocal microscopy, images were captured, revealing co-localization between the FAM-labeled DSPE-PEG-CRYYRITY and DID-labeled EVs (Fig. 2d). NTA demonstrated that the average sizes of EVs and engineered IEVs were 148.6 ± 12.9 and 163.2 ± 27 nm (Fig. 2e), respectively. The increased size of IEVs can be attributed to the peptide modification on the surface of EVs. miR-138-5p was observed to be significantly upregulated in EV-138 compared to its expression levels in EV-negative control (NC) as determined by qRT-PCR, indicating the successful loading of miR-138-5p mimic (Fig. 2f). Ultraviolet (UV) spectroscopy analysis revealed that the PFD loading efficiency, which was calculated using the standard curve, was 28.61% ± 1.2% (Supplementary Fig. 2c).

### IEVs exhibit improved CAF-targeting ability

To test the CAF-targeting ability of engineered IEVs, the cellular uptake of DID-labeled EVs by laser scanning confocal microscope (LSCM) and flow cytometry was assessed. The uptake of IEVs by CAFs was higher than that of non-targeted EVs (Fig. 2g, i, k). Similarly, we investigated the delivery efficiency of miR-138-5p (FAM) to CAFs. The FAM signal in CAFs incubated with IEVs-138 was higher than that in CAFs incubated with EVs-138, confirming that the integrin α5 peptide increased the CAF-targeting ability (Fig. 2h, j, l). qRT-PCR analysis of miR-138-3p revealed that the efficiency of IEVs to deliver the miRNA to CAFs was higher than that of non-targeted EVs (Fig. 2m). To investigate the targeting ability of IEVs in vivo, a subcutaneous fibrotic pancreatic cancer model was developed by co-implanting CAFs and PANC-1 cells into mice. The circulation time of IEVs in the peripheral blood was higher than that of undecorated EVs (Fig. 2n). Analysis with an in vivo imaging system (IVIS) revealed that the targeting ability of IEVs was higher than that of undecorated EVs (Fig. 2o). The fluorescence signal emitted by IEVs was significantly higher than that by undecorated EVs, indicating the enhanced ability of IEVs to reach pancreatic cancer site. The fluorescence intensity peaked at 4 h following the administration of EV. In addition, the kidneys, livers, lungs, spleens, hearts, and tumors were excised from mice 4 h following EV administration and subjected to ex vivo imaging. The results revealed a higher accumulation of IEVs in the tumor site compared to EVs (Fig. 2p). The tumors were sectioned to visualize the presence and spatial distribution of EVs within the tumor tissue. IEVs infiltrated the tumor tissue, which manifested as an expanded fluorescence area and enhanced fluorescence intensity when compared with undecorated EVs (Fig. 2q, Supplementary Fig. 2d).

### miR-138-5p reprograms CAF phenotype by downregulating FERMT2

**Fig. 3:**
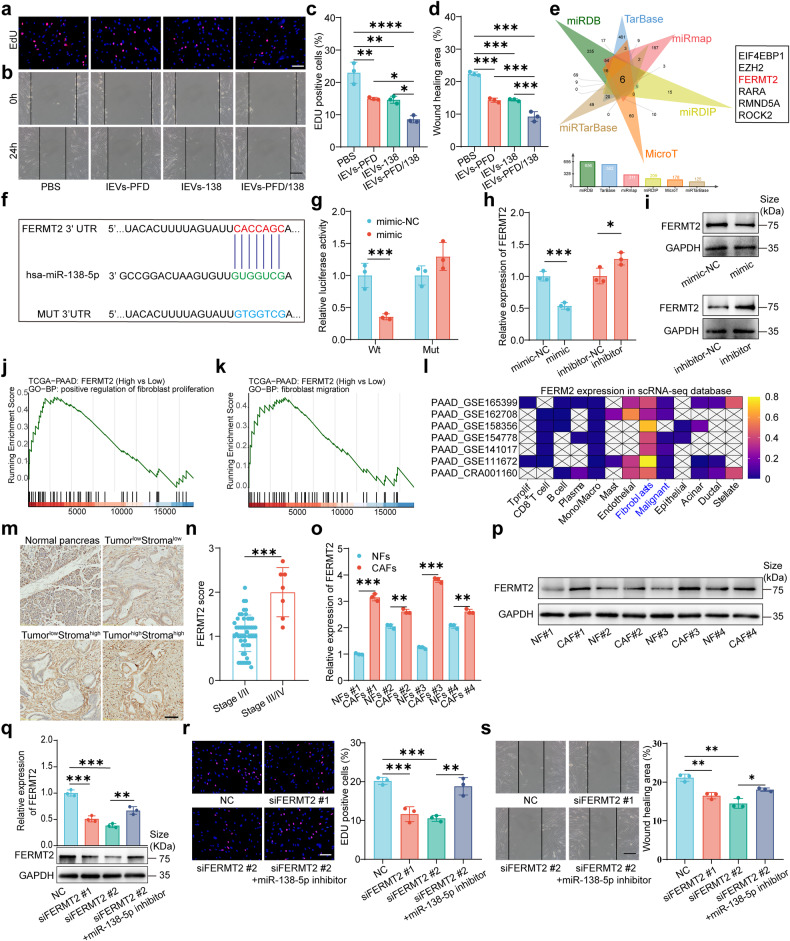
Functional and mechanistic analyses of miR-138-5p in pancreatic cancer-associated fibroblasts (CAFs). **a**–**d** EdU (Red: EdU-positive cells, blue: cell nuclei) and wound-healing assay demonstrated the inhibitory effects of extracellular vesicles (EVs) loaded with miR-138-5p and pirfenidone (PFD) and subjected to surface modification with integrin α5-targeting peptides (IEVs-PFD/138) on CAF cell proliferation and migration (scale bar = 100 μm). Data are presented as mean (± SD); *n* = 3 per group. **e** Six databases were used for predicting the potential target genes of miR-138-5p. **f** Predicted miR-138-5p-FERMT2 binding sites. **g** The results of the dual-luciferase reporter assay with miR-138-5p and FERMT2. Data are presented as mean (± SD); *n* = 3 per group. **h**, **i** Quantitative real-time polymerase chain reaction (qRT-PCR) and western blotting analyses of the impact of miR-138-5p mimic or inhibitor on the mRNA and protein levels of FERMT2 in CAFs. Data are presented as mean (± SD); *n* = 3 per group. **j**, **k** Gene set enrichment analysis of FERMT2 in pancreatic cancer. **l** Analysis of FERMT2 in pancreatic cancer with the Tumor Immune Single-cell Hub (TISCH) database (http://tisch.comp-genomics.org/home/). **m** Tissue protein microarray analysis of FERMT2 in pancreatic cancer tissues (scale bar = 100 μm). **n** Correlation of pathological scoring of FERMT2 with clinical stage in patients with pancreatic cancer. *N* = 50 for stage I/II; *n* = 7 for stage III/IV. **o**, **p** qRT-PCR and western blotting analyses of FERMT2 in primary CAFs and healthy fibroblasts (NFs). Data are presented as mean (± SD); *n* = 3 per group. **q** qRT-PCR and western blotting analyses of FERMT2 and the effect of miR-138-5p inhibitor and small interfering RNA (siRNA) against *FERMT2*. Data are presented as mean (± SD); *n* = 3 per group. **r**, **s** EdU (Red: EdU-positive cells, blue: cell nuclei) and wound-healing assay verified that miR-138-5p inhibitor mitigated the inhibitory effect of *FERMT2* knockdown on cell proliferation and migration (scale bar = 100 μm). Data are presented as mean (± SD); *n* = 3 per group

Functional experiments demonstrated that the proliferation and migration of CAFs were significantly suppressed by IEVs-PFD and IEVs-138, which produced strong inhibitory effects. Compared to other EVs, IEVs-PFD/138 had a stronger inhibition effect on CAF proliferation and migration (Fig. 3a–d, Supplementary Fig. 3a, c). These results suggest the potent anti-proliferative and anti-migratory properties of IEVs-PFD/138. Thus, IEVs-PFD/138 can modulate CAF behavior and promote TME remodeling.

To elucidate the underlying mechanism of miR-138-5p in CAFs, miR-138-5p’s potential target genes were identified by mining six databases (Fig. 3e). Among the predicted target genes, *FERMT2* exhibited the strongest correlation with CAFs according to the TIMER dataset analysis (Supplementary Fig. 3e). Thus, the miR-138-5p-FERMT2 interaction was experimentally examined. Subsequently, the binding sites between miR-138-5p and FERMT2 were predicted (Fig. 3f). The results of the dual-luciferase reporter assay illustrated the capability of miR-138-5p to bind to the wild-type FERMT2, as indicated by the notable decrease in luciferase activity (Fig. 3g). This indicates the direct miR-138-5p-FERMT2 interaction. Furthermore, transfection with the miR-138-5p mimic led to the downregulation of both mRNA (Fig. 3h) and protein (Fig. 3i) levels of FERMT2 in CAFs. Conversely, transfection with the miR-138-5p inhibitor resulted in the upregulation of both mRNA and protein levels of FERMT2 in CAFs.

The functional role of FERMT2 in pancreatic cancer was examined using gene set enrichment analysis (GSEA) with the TCGA dataset. FERMT2 expression was found to positively correlate with the proliferation and migration of CAFs (Fig. 3j, k). After analyzing the Tumor Immune Single-cell Hub (TISCH) database, it was discovered that among the cell populations in pancreatic cancer, CAFs exhibited the highest FERMT2 expression, followed by cancer cells (Figs. 3l and Supplementary Fig. 3f). This provided valuable insights into the specific cellular contexts of FERMT2 expression, indicating its potential roles in tumor-stroma interactions and the TME. The expression of FERMT2 was observed in pancreatic cancer tissues but not in healthy pancreatic tissues, as determined by tissue protein microarray analysis. Notably, the expression levels of FERMT2 in cancer tissue can be categorized into three distinct patterns (Fig. 3m). The pathological scoring of FERMT2 was positively related to the clinical stage of pancreatic cancer patients (Fig. 3n). Protein (Fig. 3p) and mRNA (Fig. 3o) expression levels of FERMT2 were significantly higher in primary CAFs compared to NFs, as determined by qRT-PCR and western blot analysis.

Next, small interfering RNAs (siRNAs) against *FERMT2* (si-FERMT2) were used to knock down the expression of *FERMT2* and determine how *FERMT2* knockdown affected the proliferative rate and migration of CAFs. In addition, the impact of FERMT2 knockdown and related phenotypes was abolished by transfecting CAFs with miR-138-5p inhibitor (Fig. 3q). The knockdown of FERMT2 considerably reduced CAF proliferation and migration, demonstrated by the EdU, CCK-8, wound-healing, and transwell assays. In addition, the miR-138-5p inhibitor effectively abrogated the *FERMT2* knockdown-mediated downregulation of the proliferation and migration of CAFs (Fig. 3r, s and Fig. s3b, d). These findings revealed the effect of *FERMT2* knockdown on CAFs and the pivotal function of miR-138-5p in mediating the inhibitory effects of *FERMT2* knockdown on the proliferation and migration of CAFs.

### FERMT2 interacts with TGFBR1 and PYCR1 in CAFs

**Fig. 4:**
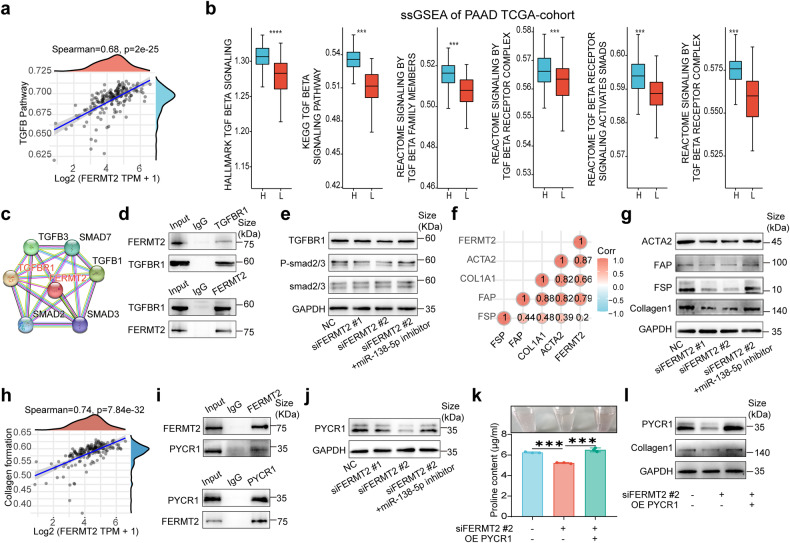
FERMT2 interacts with TGFBR1 and PYCR1 in cancer-associated fibroblasts (CAFs). **a** Correlation of FERMT2 expression with the TGF-β pathway in The Cancer Genome Atlas (TCGA) pancreatic cancer cohort. **b** Single-sample gene set enrichment analysis (ssGSEA) of FERMT2 in TCGA pancreatic cancer cohort. **c** Analysis of the interaction of FERMT2 with TGF-β signaling-related proteins using STRING database (https://cn.string-db.org/). **d** Co-immunoprecipitation (Co-IP) analysis of FERMT2 and TGFBR1. **e** Western blotting analysis of the effect of *FERMT2* knockdown and miR-138-5p inhibitor on the TGFBR1 and p-SMAD2/3 expression levels. **f** Correlation of FERMT2 with ACTA2, COL1A1, FAP, and FSP in TCGA pancreatic cancer dataset. **g** Western blotting analysis of the effect of *FERMT2* knockdown and miR-138-5p inhibitor on the ACTA2, collagen1, FAP, and FSP expression levels. **h** Correlation of FERMT2 expression with the collagen production pathway in TCGA pancreatic cancer dataset. **i** Co-IP analysis of FERMT2 and TGFBR1. **j** Western blotting analysis of the impact of *FERMT2* knockdown and miR-138-5p inhibitor on PYCR1 expression. **k** Effect of *FERMT2* knockdown and PYCR1 overexpression on the proline content. Data are presented as mean (±SD); *n* = 3 per group. **l** Western blotting analysis of the effect of *FERMT2* knockdown and PYCR1 overexpression on the PYCR1 and collagen1 expression levels

Analysis of the TCGA pancreatic cancer dataset revealed that FERMT2 was positively correlated with the TGF-β signaling pathway (Fig. 4a). In addition, single-sample GSEA (ssGSEA) confirmed the positive correlation between FERMT2 and the TGF-β signaling pathway (Fig. 4b). Furthermore, analysis using the STRING database indicated that FERMT2 can potentially interact with TGFBR1 (Fig. 4c). This interaction was experimentally validated using co-IP analysis (Fig. 4d). Western blotting demonstrated that the protein expression levels of TGFBR1 and phosphorylated SMAD2/3 (p-SMAD2/3) were downregulated upon *FERMT2* knockdown and restored upon miR-138-5p inhibition (Fig. 4e). Besides, the mRNA expression of FERMT2 positively correlated with ACTA2, COL1A1, FAP, and FSP based on TCGA pancreatic cancer data. (Fig. 4f). Western blotting results revealed that the protein expression levels of ACTA2, collagen 1, FAP, and FSP were decreased upon FERMT2 knockdown and restored upon the inhibition of miR-138-5p (Fig. 4g). These findings demonstrated that the inhibitory impact of miR-138-5p on the TGF-β signaling pathway could counteract the inhibitory effects of *FERMT2* knockdown, indicating a regulatory function of miR-138-5p in the FERMT2-TGFBR1-TGF-β signaling pathway, which can further regulate CAF phenotype and collagen formation.

FERMT2 was positively correlated with the collagen formation pathway (Fig. 4h). PYCR1, a key enzyme involved in proline metabolism, regulates collagen deposition and remodeling. Co-IP experiments revealed the interaction between FERMT2 and PYCR1, indicating that these two proteins can form a complex (Fig. 4i). In addition, miR-138-5p inhibitor mitigated the *FERMT2* knockdown-mediated downregulation of PYCR1 levels, as shown by Western blotting analysis (Fig. 4j). *FERMT2* knockdown downregulated the proline levels. Conversely, PYCR1 overexpression upregulated the proline levels (Fig. 4k). Western blotting analysis revealed that PYCR1 overexpression mitigated the *FERMT2* knockdown-mediated downregulation of PYCR1 and COL1A1 (Fig. 4l). Based on these data, the inhibition effect of miR-138-5p on PYCR1 expression can counteract the inhibitory effects of *FERMT2* knockdown, indicating a regulatory function of miR-138-5p in modulating the FERMT2-PYCR1 axis, which can further regulate proline and collagen formation.

### IEVs-PFD/138 treatment reverses CAF activation in vitro

**Fig. 5:**
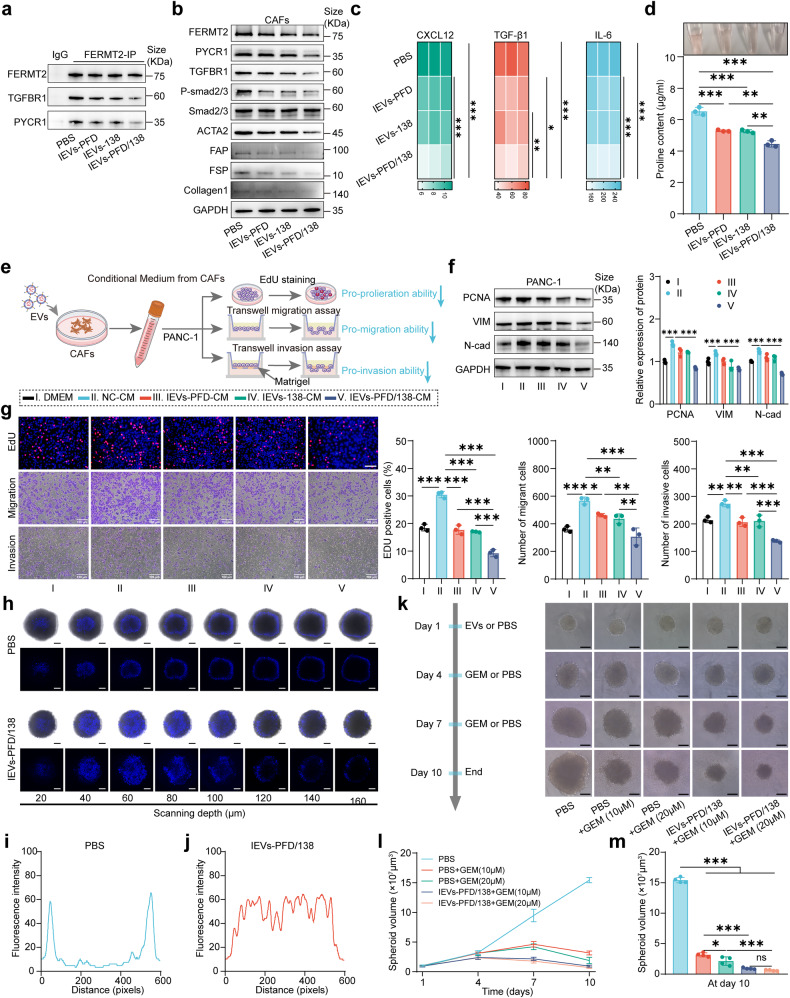
Role of extracellular vesicles (EVs) loaded with miR-138-5p and pirfenidone (PFD) and subjected to surface modification with integrin α5-targeting peptides (IEVs-PFD/138) in cancer-associated fibroblast (CAF) phenotype reversal, cancer cell inhibition, and enhanced drug penetration. **a** Immunoprecipitation (IP) analysis of the FERMT2-TGFBR1 and FERMT2-PYCR1 complexes. **b** Western blotting analysis of the expression levels of FERMT2, PYCR1, TGFBR1, p-SMAD2/3, ACTA2, FAP, FSP, and collagen1. **c** Enzyme-linked immunosorbent assay (ELISA) for analyzing the secretion of IL6, TGFB1, and CXCL12 by CAFs. Data are presented as mean (± SD); *n* = 3 per group. **d** Proline levels in CAFs. Data represent the mean (± SD); *n* = 3 per group. **e** Scheme of the effect of conditioned medium (CM) derived from EV-treated CAFs on PANC-1 cells. **f** Western blotting analysis of PCNA, VIM, and N-cad in PANC-1 cells treated with CM derived from EV-treated CAFs. Data are presented as mean (±SD); *n* = 3 per group. **g** Evaluation of the proliferation, migration, and invasion of PANC-1 cells treated with CM derived from CAFs treated with EVs (Red: EdU-positive cells, blue: cell nuclei), (scale bar = 100 μm). Data are presented as mean (±SD); *n* = 3 per group. **h**–**j** Enhanced fluorescence intensity and penetration depth of Hoechst 33258 in stroma-rich three-dimensional (3D) multicellular tumor spheroids treated with IEVs-PFD/138 (scale bar = 100 μm). **k**–**m** Comparison of 3D multicellular tumor spheroid volume after treatment with the combination of IEVs-PFD/138 and gemcitabine (GEM) (scale bar = 100 μm). Data represent the mean (± SD); *n* = 3 per group

The immunoprecipitation (IP) assay results demonstrated that IEVs-PFD and IEVs-138 decreased the abundance of the FERMT2-TGFBR1 and FERMT2-PYCR1 complexes. IEVs-PFD/138 exerted the highest inhibitory effects on the formation of these complexes (Fig. 5a). Western blotting analysis demonstrated that IEVs-PFD and IEVs-138 effectively downregulated the expression levels of FERMT2, PYCR1, and TGFBR1 (Fig. 5b), as well as the expression levels of p-SMAD2/3, indicating the suppression of TGF-β signaling pathway activity (Fig. 5b). In addition, the levels of ACTA2, FAP, FSP, and collagen1 were considerably downregulated by IEVs-PFD and IEVs-138 (Fig. 5b). Compared to other EVs, IEVs-PFD/138 had greater inhibitory impacts on the expression of these proteins. Both IEVs-PFD and IEVs-138 effectively suppressed CAF secretion of IL-6, TGFB1, and CXCL12, according to the results of the enzyme-linked immunosorbent assay (ELISA) (Fig. 5c). In particular, IEVs-PFD/138 exerted the highest inhibitory effects on the secretion of IL6, TGFB1, and CXCL12. These findings suggest that IEVs-PFD/138 suppressed the production of pro-inflammatory and pro-tumorigenic factors in CAFs. Analysis of the proline levels revealed that IEVs-PFD and IEVs-138 effectively downregulated the intracellular proline content in CAFs (Fig. 5d). In particular, IEVs-PFD/138 exerted the highest inhibitory effects on the proline levels. These findings suggest that IEVs-PFD/138 modulated proline metabolism in CAFs and consequently downregulated intracellular proline content. Thus, the application of IEVs-PFD/138 is a potential therapeutic strategy for modulating the activation and secretory profile of CAFs and the formation of collagen.

### IEVs-PFD/138 treatment suppresses the tumor-promoting effect of CAFs in vitro

To elucidate the effect of EV-treated CAFs on the biological behaviors (proliferation, migration, and invasion) of pancreatic cancer cells, PANC-1 cells were incubated with the conditioned medium (CM) of EV-treated CAFs (Fig. 5e). After subjecting PANC-1 cells to CM treatment, Western blotting analysis showed an increase in the levels of PCNA, VIM, and N-cadherin. However, the application of CM derived from EV-treated CAFs, specifically IEVs-PFD and IEVs-138, attenuated this effect. Notably, the inhibitory effect was most pronounced with the application of IEVs-PFD/138 (Fig. 5f). The results of the EdU, transwell migration, and invasion assays revealed that the CM of CAFs the capacity of PANC-1 cells to proliferate, migrate, and invade. In contrast, the CM of CAFs treated with EVs, especially IEVs-PFD and IEVs-138, suppressed the capacity of PANC-1 cells to proliferate, migrate, and invade. Treatment with the CM of CAFs treated with IEVs-PFD/138 exerted the highest inhibitory effect on the proliferation, migration, and invasion of PANC-1 cells (Fig. 5g). These findings revealed the regulatory effects of CAF-derived CM on the proliferation, migration, and invasion of PANC-1 cells. Overall, the potent inhibitory impact of IEVs-PFD/138 improved our understanding of the reciprocal interactions between pancreatic cancer cells and CAFs and can aid in developing novel strategies targeting cancer progression.

### IEVs-PFD/138 treatment enhances drug penetration into stroma-rich 3D multicellular tumor spheroids

The dense ECM within the desmoplastic pancreatic cancer TME is a major obstacle to the penetration of anticancer therapeutics. To address this challenge, a stroma-rich 3D multicellular tumor spheroid model was established by co-culturing PANC-1 cells with CAFs. This model closely mimics the desmoplastic characteristics of the pancreatic cancer TME. The effect of IEVs-PFD/138 on the stromal components and the penetration of small-molecule chemotherapy drugs in stroma-rich 3D multicellular tumor spheroids was examined. To simulate the penetration of small-molecule drugs, Hoechst 33258, a commonly used fluorescence probe was applied. IEVs-PFD/138-treated 3D multicellular tumor spheroids exhibited enhanced Hoechst 33258 fluorescence intensity and penetration depth (Fig. 5h–j). To validate the effect of IEVs-PFD/138 on drug penetration, 3D multicellular tumor spheroids were co-treated with IEVs-PFD/138 and GEM. At equivalent concentrations of GEM (10 and 20 μM), the volume of 3D spheroids pre-treated with IEVs-PFD/138 was significantly reduced relative to that of 3D spheroids pre-treated with phosphate-buffered saline (PBS) (Fig. 5k–m). Furthermore, necrotic cells were observed surrounding the 3D multicellular tumor spheroids. These findings suggested that IEVs-PFD/138 potentiate the efficacy of small-molecule anti-tumor drugs in the TME of pancreatic cancer by abrogating the buildup of fibrotic stroma and improving drug delivery.

### IEVs-PFD/138 treatment reduces activated CAFs and proliferation in 3D human PDAC tumor explants

EVs were introduced into 3D human PDAC tumor explants derived from surgical resections, followed by seven days of continuous culture (Supplementary Fig. 4a). This model maintains the 3D organization and interactions of PDAC cells and stromal cells in a tissue culture dish. IEVs-PFD/138 exhibited a significant decrease in activated CAFs and cellular proliferative rate compared to the control groups (Supplementary Fig. 4b, c).

### IEVs-PFD/138 treatment exerts anti-tumor effects and reverses CAF activation in a subcutaneous model of desmoplastic pancreatic cancer

**Fig. 6:**
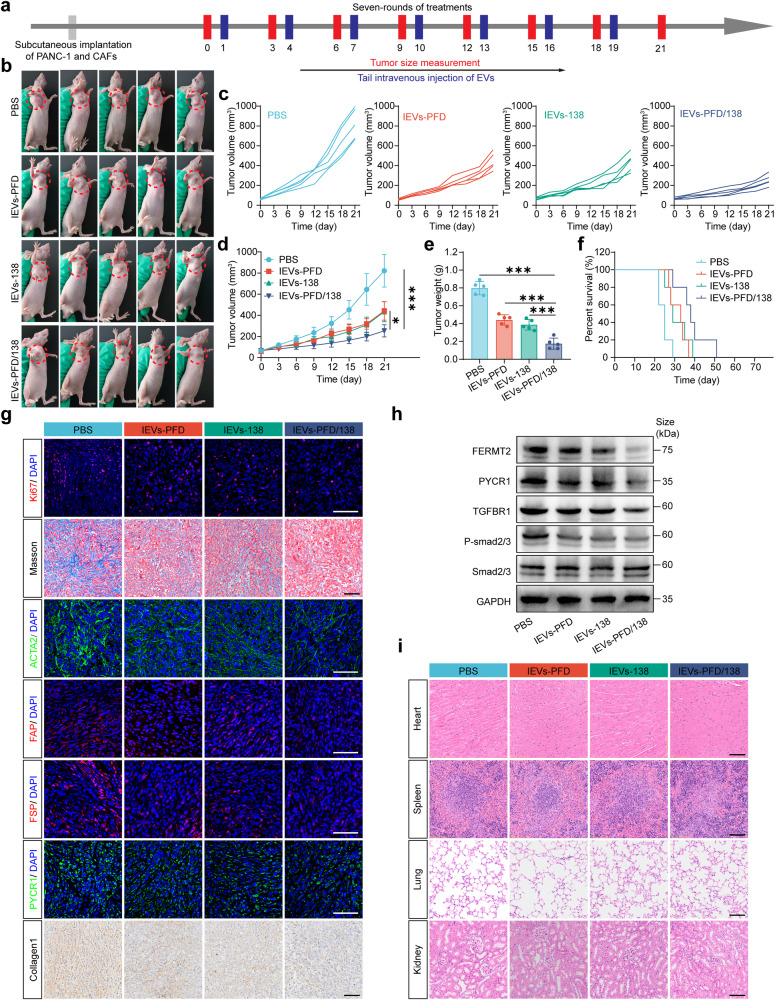
Effect of treatment with extracellular vesicles (EVs) loaded with miR-138-5p and pirfenidone (PFD) and subjected to surface modification with integrin α5-targeting peptides (IEVs-PFD/138) in the subcutaneous desmoplastic pancreatic cancer model. **a** Scheme of IEVs-PFD/138 treatment. **b** Images of the mouse model after treatment on day 21. **c**, **d** Tumor growth curves of the mouse models. Data are presented as mean (± SD); *n* = 5 per group. **e** Tumor weight of the mouse models. Data are presented as mean (± SD); *n* = 5 per group. **f** Effect of treatment on the survival of the mouse model. **g** Ki67 immunofluorescence staining, Masson’s staining, ACTA2 immunofluorescence staining, FAP immunofluorescence staining, FSP immunofluorescence staining, PYCR1 immunofluorescence staining, and collagen1 immunohistochemical staining of tumors from mice under different treatments (scale bar = 100 μm). **h** Western blotting analysis of the FERMT2, PYCR1, TGFBR1, and p-SMAD2/3 expression levels in tumors from mice administered various treatments. **i** Hematoxylin and eosin (HE) staining of vital organs from mice administered various treatments (scale bar = 100 μm)

To examine the therapeutic implications and fundamental mechanisms of IEVs-PFD/138 in vivo, a model of subcutaneous desmoplastic pancreatic cancer was developed in mice via the co-transplantation of PANC-1 cells and CAFs. Subsequently, mice were intravenously injected with EVs or PBS for seven treatment cycles (Fig. 6a). The results demonstrated a significant inhibition of tumor growth following treatment with both IEVs-PFD and IEVs-138 (Fig. 6b–e). Remarkably, IEVs-138/PFD exhibited a pronounced anti-tumor effect, leading to a significant extension in the survival time of nude mice (time required for the tumor volume to reach 1000 mm^3^) (Fig. 6d).

Immunofluorescence analysis revealed that tumor cell proliferation was significantly downregulated in the IEVs-PFD/138-treated group as evidenced by the decreased immunofluorescence intensity of the proliferation marker Ki67 (Fig. 6g). The immunofluorescence intensities of ACTA2, FAP, and FSP, which serve as activation markers for CAFs, were markedly downregulated in the IEVs-PFD/138-treated group, indicating the reversal of CAF activation (Fig. 6g). In addition, the collagen content of the tumors decreased considerably in the group that received IEVs-PFD/138, as evidenced by Masson’s trichrome staining. Moreover, the PYCR1 and collagen1 levels were downregulated in the IEVs-PFD/138-treated group, suggesting that treatment with IEVs-PFD/138 decreases the ability of CAFs to produce collagen (Fig. 6g). Western blotting analysis revealed the downregulation of FERMT2 and PYCR1 in the IEVs-PFD/138-treated group (Fig. 6h). In addition, the TGFBR1 expression level was significantly downregulated in the IEVs-PFD/138-treated group. Furthermore, the p-SMAD2/3 levels were downregulated in the IEVs-PFD/138-treated group, indicating the suppression of the TGF-β signaling pathway (Fig. 6h). Furthermore, histological examination of vital organs using hematoxylin and eosin (HE) staining demonstrated no significant damage in important organs of nude mice treated with EVs (Fig. 6i). This indicates that EVs do not exert toxic effects on vital organs. Thus, IEVs-PFD/138 treatment exerts inhibitory effects on pancreatic cell proliferation and the TGF-β signaling pathway, reverses CAF activation, and downregulates collagen deposition. These findings provide valuable insights into the mechanisms governing the anti-tumor and anti-fibrotic properties of IEVs-PFD/138 and suggest that IEVs-PFD/138 treatment is a promising treatment strategy for pancreatic cancer.

### IEVs-PFD/138 treatment enhances the therapeutic efficacy of GEM and reverses desmoplastic TME phenotype in a pancreatic cancer PDX model

**Fig. 7:**
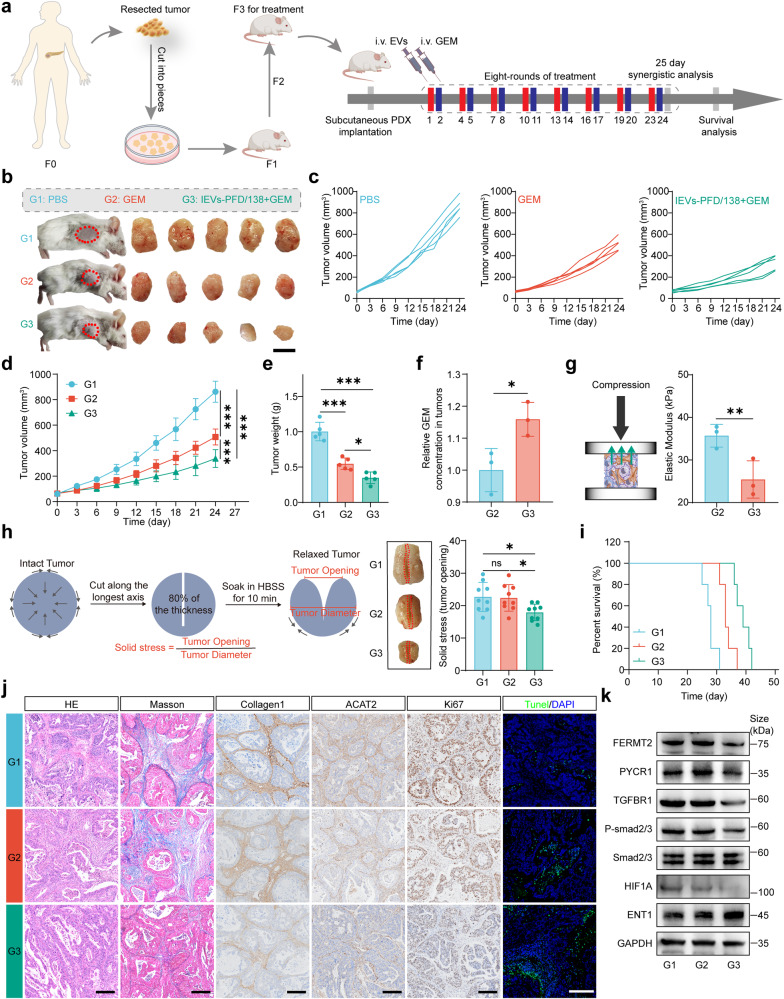
Effect of treatment with extracellular vesicles (EVs) loaded with miR-138-5p and pirfenidone (PFD) and subjected to surface modification with integrin α5-targeting peptides (IEVs-PFD/138) in the patient-derived xenograft (PDX) pancreatic cancer models. **a** Scheme of PDX model establishment and treatment. **b** Images of mouse models and tumors after treatment on day 24. **c**, **d** Tumor growth curves of the mouse models. Data are presented as mean (± SD); *n* = 5 per group. **e** Tumor weight of the mouse models. Data are presented as mean (±SD); *n* = 5 per group. **f** Relative gemcitabine (GEM) concentrations in tumors. Data are presented as mean (±SD); *n* = 3 per group. **g** Elastic modulus of tumors. Data are presented as mean (± SD); *n* = 3 per group. **h** Solid stress of tumors (three tumors with each tumor having three parts). **i** Effect of treatment on the survival of the mouse model. **j** Hematoxylin and eosin (HE) staining, Masson’s staining, collagen1 immunohistochemical staining, ACTA2 immunohistochemical staining, Ki67 immunohistochemical staining, and Tunel staining of tumors from mice administered various treatments (scale bar = 200 μm). **k** Western blotting analysis of the FERMT2, PYCR1, TGFBR1, P-SMAD2/3, HIF1A, and ENT1 expression levels in tumors from mice subjected to different treatments

The PDX model is considered a clinically relevant model as it utilizes patient tumor samples for transplantation into immunodeficient mice, preserving the tumor heterogeneity and stromal microenvironment. In this study, PDX at the F3 passage was used as the experimental model. EVs and GEM were sequentially administered via the tail vein for eight treatment cycles (Fig. 7a). On day 25, tumors were collected from five mice in each group for further analysis. Notably, the tumor growth rate was found to be considerably reduced in the GEM-treated group compared to the PBS-treated group, as indicated by the tumor volume growth curve analysis. The tumor growth rate was the slowest in the group treated with the combination of IEVs-PFD/138 and GEM (Fig. 7b–d). In addition, tumors treated with GEM had a reduced weight relative to those treated with PBS. The tumor weight was the lowest in the group treated with the combination of IEVs-PFD/138 and GEM (Fig. 7e).

IEVs-PDF/138 suppressed tumor stroma generation, enhancing drug penetration. Thus, this study quantified GEM content in tumor tissues using UV spectroscopy. In comparison to the GEM-treated group, the IEVs-PDF/138-treated group exhibited reduced tumor concentrations of GEM (Fig. 7f). The tumor elastic modulus was significantly downregulated in the IEVs-PFD/138-treated group (Fig. 7g). Furthermore, the effect of IEVs-PFD/138 on the solid stress of tumors was examined by measuring solid stress levels according to an established protocol. Significantly reduced solid stress was observed within tumors subjected to IEVs-PFD/138 as compared to the groups treated with PBS and GEM (Fig. 7h). These findings suggest that IEVs-PFD/138 treatment effectively decreases tumor stroma generation. The observed reduction in elastic modulus and solid stress indicated a modulation of the mechanical forces exerted by the stromal components within the TME. The desmoplastic stroma in pancreatic cancer is reported to generate physical barriers that limit drug penetration and hinder therapeutic efficacy. The observed reduction in elastic modulus and solid stress within the tumors induced by IEVs-PFD/138 treatment can explain the enhanced efficiency of GEM. The reduction in solid stress alleviated the mechanical compression exerted on blood vessels and enhanced vascular perfusion within the tumor, facilitating the delivery of GEM to tumor cells. In addition, the decreased stromal density and interstitial fluid pressure resulting from stroma reduction may contribute to enhanced drug diffusion and distribution throughout the tumor. Survival analysis indicated that mice in the EV-treated group exhibited the longest survival time (time required for the tumor volume to reach 1000 mm^3^) (Fig. 7i). These findings suggest the potential of IEVs-PFD/138 in synergistically enhancing the therapeutic efficacy of GEM in the PDX model.

HE staining, Masson’s staining, and immunohistochemical (IHC) analysis of collagen1, revealed that IEVs-PFD/138 markedly downregulated tumor collagen deposition, indicating effective modulation of the ECM components (Fig. 7j). In addition, IHC analysis revealed the downregulation of ACTA2 expression, indicating the suppression of CAF activation. These findings suggest that IEVs-PFD/138 treatment suppresses CAF activation, contributing to the modulation of the TME (Fig. 7j). The expression of KI67 was markedly downregulated in the IEVs-PFD/138-treated group (Fig. 7j), suggesting the decreased proliferative capacity of tumor cells. In addition, TUNEL staining revealed that IEVs-PFD/138 significantly upregulated the number of apoptotic cells (Fig. 7j). This further supports the anti-proliferative and pro-apoptotic effects of IEVs-PFD/138. These results can be attributed to the enhanced efficacy of GEM when combined with IEVs-PFD/138.

The FERMT2 and PYCR1 protein expression were found to be decreased in the group that received IEVs-PFD/138, as determined by Western blotting analysis (Fig. 7k). In addition, IEVs-PFD/138 downregulated the TGFBR1 and p-SMAD2/3 levels, which suggests inhibition of the TGF-β signaling pathway (Fig. 7k). The TGF-β pathway assumes a multifaceted function in cancer as it can promote CAF activation and exert tumorigenic effects. The downregulation of TGFBR1 and p-SMAD2/3 suggests the suppression of TGF-β signaling, which may contribute to the reversal of CAF activation and the inhibition of cancer-promoting effects. HIF1A expression was decreased in the group treated with IEVs-PFD/138 (Fig. 7k). The downregulation of HIF1A, an essential regulator of hypoxia-induced cellular response, indicates the alleviation of tumor hypoxia. The alleviation of hypoxia can be attributed to the IEVs-PFD/138-mediated downregulation of tumor stromal pressure. The downregulation of HIF1A suggests a shift toward a less hypoxic TME. Moreover, the IEVs-PFD/138 treatment resulted in increased expression of ENT1 protein in tumor tissue (Fig. 7k). ENT1 is a nucleoside transporter responsible for the uptake of GEM by cancer cells. The upregulation of ENT1 expression suggests enhanced GEM uptake by pancreatic cancer cells, leading to greater drug sensitivity. The increased ENT1 expression, influenced by the modulation of the TGF-β pathway and HIF1A, facilitates increased intracellular GEM accumulation and improved therapeutic response.

These findings have significant implications for the development of innovative therapeutic approaches for pancreatic cancer. The suppression of CAF activation and collagen deposition induced by IEVs-PFD/138 treatment may effectively alleviate the desmoplastic stromal barrier associated with tumor progression and therapy resistance. Furthermore, the enhanced efficacy of GEM in combination with IEVs-PFD/138 highlights the potential synergistic effects of this treatment strategy for pancreatic cancer.

### IEVs-PFD/138 treatment potentiates the therapeutic efficacy of GEM and inhibits metastasis in an orthotopic model of desmoplastic pancreatic cancer

**Fig. 8:**
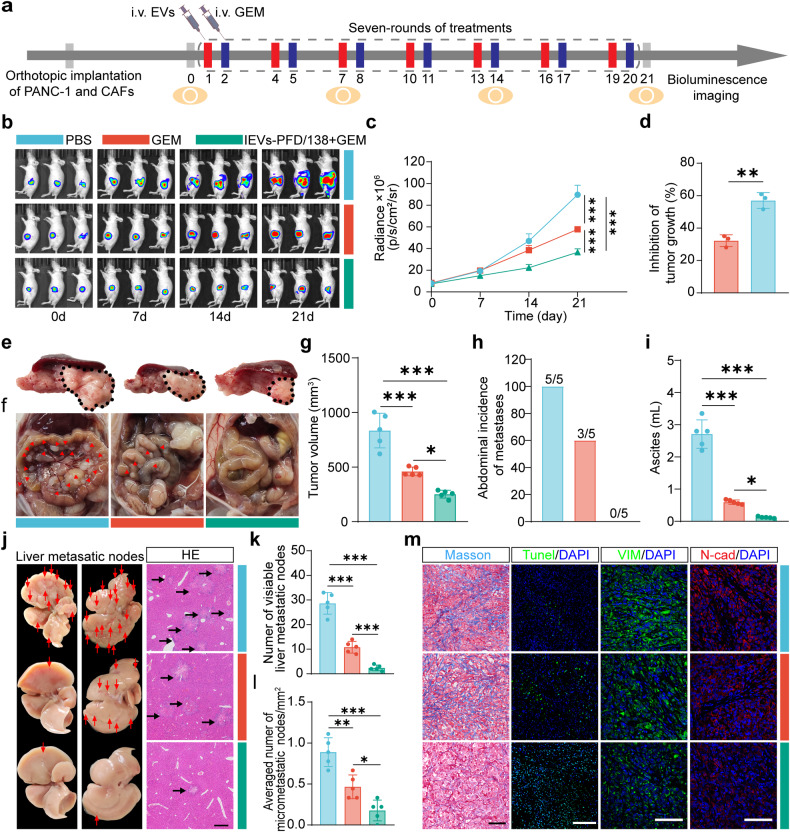
Effects of treatment with extracellular vesicles (EVs) loaded with miR-138-5p and pirfenidone (PFD) and subjected to surface modification with integrin α5-targeting peptides (IEVs-PFD/138) in the orthotopic desmoplastic pancreatic cancer models. **a** Scheme of IEVs-PFD/138 and gemcitabine (GEM) treatment. **b** Illustrative in vivo bioluminescence images of mice. **c** Bioluminescence intensity quantification of mice with orthotopic pancreatic tumors. Data represent the mean (±SD); *n* = 3 per group. **d** Growth-inhibitory effects on tumor based on bioluminescence intensity. Data are presented as mean (± SD); *n* = 3 per group. **e** Images of excised tumors with spleen and pancreas in different treatment groups on day 21. **f** Images of abdominal metastases. **g** Tumor volume. Data are presented as mean (± SD); *n* = 5 per group. **h** Incidence of abdominal metastases. **i** Amount of ascitic fluid. Data are presented as mean (±SD); *n* = 3 per group. **j** Images of excised livers and hematoxylin and eosin (HE) staining of liver metastasis (scale bar = 500 μm). **k** Number of visible liver metastatic nodes. Data are presented as mean (± SD); *n* = 3 per group. **l** Average number of micro-metastatic nodes/mm^2^. Data are presented as mean (± SD); *n* = 3 per group. **m** Masson’s staining (scale bar = 100 μm), Tunel (scale bar = 200 μm), VIM immunofluorescence (scale bar = 100 μm) and N-cad immunofluorescence (scale bar = 100 μm) staining of tumors

The orthotopic model of desmoplastic pancreatic cancer established using PANC-1 cells and CAFs accurately reflects the native TME and anatomical characteristics of the primary tumor site. This model enables the evaluation of cancer-stroma interactions and metastatic dissemination. In addition, this model provides a comprehensive representation of tumor biology and therapeutic response. IEVs-PFD/138 and GEM were sequentially and intravenously administered for seven treatment cycles. Bioluminescence imaging was performed on days 0, 7, 14, and 21 to monitor the growth of the orthotopic pancreatic tumors (Fig. 8a). The group that received GEM treatment exhibited a reduced growth rate of pancreatic tumors compared to the group that received PBS. The combination of GEM and IEVs-PFD/138 significantly decreased tumor growth (Fig. 8b–d). These findings suggest the potential of IEVs-PFD/138 in enhancing the therapeutic efficacy of GEM against orthotopic pancreatic cancer.

On day 21, all nude mice were euthanized and subjected to necropsy. In the GEM-treated group, the volume of the tumor was considerably reduced in comparison to the PBS-treated group. Meanwhile, the tumor volume was the lowest in the group treated with the combination of IEVs-PFD/138 and GEM (Fig. 8e, g). Furthermore, none of the mice in the IEVs-PFD/138-treated group exhibited macroscopically visible peritoneal metastasis, whereas peritoneal metastases were observed macroscopically in all five mice, which were treated with PBS, and in three mice, which were treated with GEM (Fig. 8f, h). In addition, analysis of abdominal metastasis revealed the accumulation of ascitic fluid in the peritoneal cavity of mice in the PBS-treated group. In contrast, the group that received GEM treatment had a reduced quantity of ascitic fluid compared to the group that received PBS. Only a minimal amount of ascitic fluid was observed in the group treated with the combination of IEVs-PFD/138 and GEM (Fig. 8i). Macroscopically visible metastatic nodules were observed on the liver surface in the PBS-treated group. The incidence of macroscopically visible liver metastasis was suppressed in the GEM-treated mice and further attenuated in the group treated with the combination of IEVs-PFD/138 and GEM (Fig. 8j, k). Moreover, The histological analysis confirmed that the number of microscopic nodules was comparatively reduced in the GEM-treated mice as opposed to those that received PBS-treated. The group treated with the combination of IEVs-PFD/138 and GEM group exhibited the least number of microscopic nodules (Fig. 8j, l). These data illustrate that combining IEVs-PFD/138 and GEM decreases tumor volume and attenuates metastasis in the experimental model of desmoplastic pancreatic cancer. Consistent with the findings in subcutaneous tumor implantation and PDX models, Masson’s staining revealed that the collagen content of the mice treated with IEVs-PFD/138 was found to be comparatively lower than that of the mice treated with PBS and GEM, suggesting the inactivation of CAFs and the inhibition of collagen secretion (Fig. 8m). TUNEL staining revealed that the number of apoptotic cells in the mice that received GEM was increased relative to those that received PBS. Treatment with the combination of IEVs-PFD/138 and GEM further increased the number of apoptotic cells (Fig. 8m). Immunofluorescence staining for N-cad and VIM showed that GEM reduced the migratory capacity of pancreatic cancer cells, with a more pronounced effect observed in combination with IEVs-PFD/138 (Fig. 8m). These results indicated that IEVs-PFD/138 treatment downregulates collagen content through the inactivation of CAFs and the inhibition of collagen secretion. Furthermore, TUNEL staining revealed that the combination of IEVs-PFD/138 and GEM enhances apoptotic cell death. In addition, GEM treatment reduces the migratory ability of pancreatic cancer cells, and this effect is further potentiated by the addition of IEVs-PFD/138, consistent with the observed reduction in peritoneal metastasis.

### IEVs-PFD/138 treatment enhances the therapeutic efficacy of GEM in an orthotopic immunocompetent model of desmoplastic pancreatic cancer

**Fig. 9:**
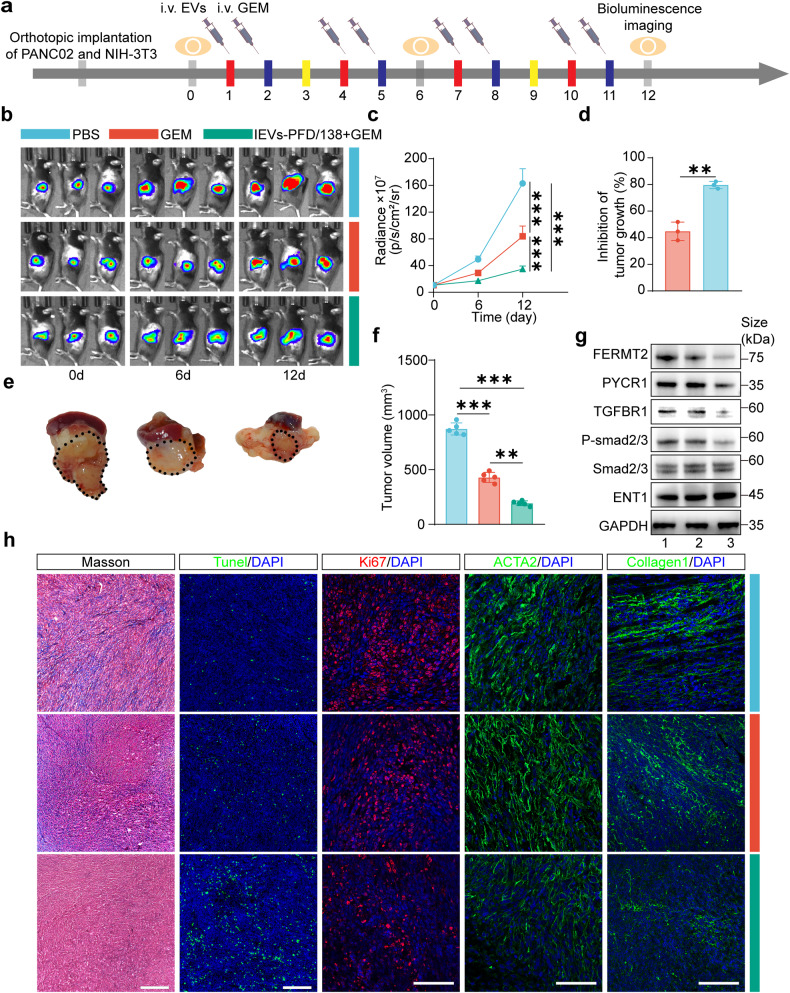
Effects of treatment with extracellular vesicles (EVs) loaded with miR-138-5p and pirfenidone (PFD) and subjected to surface modification with integrin α5-targeting peptides (IEVs-PFD/138) in the orthotopic desmoplastic pancreatic cancer C57/B6 models. **a** Scheme of IEVs-PFD/138 and gemcitabine (GEM) treatment. **b** Sample in vivo bioluminescence images of mice. **c** Bioluminescence intensity quantification of mice with orthotopic pancreatic tumors. Data represent the mean (± SD); *n* = 3 per group. **d** Growth-inhibitory effects on tumor based on bioluminescence intensity. Data represent the mean (±SD); *n* = 3 per group. **e** Images of excised tumors with spleen and pancreas in different treatment groups on day 12. **f** Tumor volume. Data are presented as mean (±SD); *n* = 5 per group. **g** Western blotting analysis of the FERMT2, PYCR1, TGFBR1, P-SMAD2/3, and ENT1 expression levels in tumors from mice subjected to different treatments. **h** Masson’s staining (scale bar = 100 μm), Tunel (scale bar = 200 μm), KI67 immunofluorescence (scale bar = 100 μm), ACTA2 immunofluorescence (scale bar = 100 μm), and collagen1 immunofluorescence (scale bar = 100 μm) staining of tumors

IEVs-PFD/138 and GEM were administered intravenously across four treatment cycles in an immunocompetent mouse model of orthotopic pancreatic cancer (Fig. 9a). Bioluminescence imaging conducted on days 0, 6, and 12 exhibited a notable reduction in tumor growth in the GEM-treated group, with a further decline observed in the combination-treated group of IEVs-PFD/138 and GEM (Fig. 9b–d). On day 12, all mice were euthanized for necropsy. The tumor volume in the mice treated with GEM was found to be significantly decreased relative to that in the mice that received PBS. Notably, the group treated with the combination of IEVs-PFD/138 and GEM displayed the lowest tumor volume. (Fig. 9e, f). This underscores the significant potential of IEVs-PFD/138 to augment the therapeutic effectiveness of GEM. Western blot analysis revealed a downregulation of FERMT2 and PYCR1 in the IEVs-PFD/138-treated group, coupled with the inhibition of the TGF-β signaling pathway. The increased expression of ENT1 suggested heightened sensitivity to GEM (Fig. 9g). Masson’s staining revealed lower collagen content in the IEVs-PFD/138-treated group, indicating the inactivation of CAFs and the inhibition of collagen secretion (Fig. 9h). TUNEL staining showed increased apoptotic cells in the GEM-treated group, with further enhancement in the IEVs-PFD/138 and GEM combination-treated group (Fig. 9h). KI67 expression was markedly downregulated in the IEVs-PFD/138-treated group, suggesting decreased proliferative capacity of tumor cells. Immunofluorescence analysis revealed downregulation of ACTA2 and collagen1 expression, indicating suppression of CAF activation. These findings collectively suggest that IEVs-PFD/138 treatment effectively suppresses CAF activation, contributing to the modulation of the tumor microenvironment (Fig. 9h).

## Discussion

Pancreatic cancer is hallmarked by highly fibrotic desmoplastic stroma and is generated by CAFs. CAFs exert regulatory effects on cancer cell behavior by modulating ECM deposition and remodeling, cytokine secretion, and interactions with cancer cells. Thus, CAFs are implicated in the carcinogenesis, progression, and metastasis of cancer. Furthermore, CAFs regulate pancreatic cancer therapy response as they contribute to therapeutic resistance through diverse mechanisms. The presence of CAFs poses a significant obstacle to pancreatic cancer treatment. Therefore, the development of targeted therapeutic approaches against CAFs is critical for pancreatic cancer treatment. This study constructed engineered EVs that were surface-modified with integrin α5-targeting peptide and loaded with miR-138-5p and the anti-fibrotic drug PFD. Engineered IEVs-PFD/138 reprogrammed pancreatic CAFs and reversed their tumor-promoting effects. In addition, the IEVs-PFD/138-induced downregulation of ECM deposition decreased tumor pressure, enhancing the penetration of GEM. Furthermore, the IEVs-PFD/138-induced alteration of the TME contributed to the increased sensitivity of cancer cells to GEM and potentiated the therapeutic efficacy of GEM.

In this study, pancreatic CAFs were shown to have downregulated levels of miR-138-5p. The ability of miR-138-5p to reverse CAF activation was confirmed in functional studies. Existing literature has reported that miR-138-5p can inhibit the progression of various tumors^9,16–18^ and reverse the fibrotic progression of various diseases.^11,19^ Therefore, miR-138-5p is a promising target for reprogramming pancreatic CAFs. PFD is a first-line medication used for the treatment of IPF.^20^ In this study, PFD was co-loaded with miR-138-5p into engineered EVs to reprogram pancreatic CAFs. Previous studies have reported that PFD suppresses desmoplasia of pancreatic cancer by regulating pancreatic stellate cells (PSCs).^14^ PFD exerts therapeutic effects by downregulating ECM components, reducing tumor pressure, and relieving compression on tumor blood vessels. This process leads to an increased density of functional blood vessels, improving oxygen supply within the tumor.^21^

BMSC-EVs were loaded with miR-138 and PFD and subjected to surface modification with integrin α5-targeting peptide. The peptide modification enhances the targeting specificity of EVs. For example, EVs modified with angiopep-2 and transferrin peptide can traverse the blood-brain barrier and reach gliomas.^22^ The phospholipid membrane anchor technology was used to modify the EV membrane. Due to the high expression of integrin α5 on the membrane of pancreatic cancer CAFs, we designed a DSPE-PEG-integrin α5 peptide to modify the surface of BMSCs-EVs. The surface modification with the integrin α5-targeting peptide is a thoughtful design choice as it enhances the specificity of EVs for pancreatic CAFs. In addition to improving the selectivity of EVs, this modification minimizes off-target effects on non-cancer cells, contributing to the safety and precision of the therapeutic approach. Engineered EVs exerted growth-inhibitory effects on pancreatic cancer. However, the potential heterogeneity in treatment responses among different patients cannot be ruled out. Therefore, comprehensive studies must be performed to improve our understanding of potential variations in the efficacy of this treatment approach across diverse patient cohorts.

Mechanistically, the levels of FERMT2 mRNA and protein can be decreased when miR-138-5p binds to the 3’-UTR of FERMT2. Research has previously demonstrated that TGF-β1 upregulates FERMT2 expression in pancreatic cancer cells. Subsequently, FERMT2 promotes pancreatic cancer progression by downregulating HOXB9 and E-cadherin. In addition, FERMT2 upregulates TGFBR1 expression, forming a positive feedback loop with the TGF-β signaling pathway.^23^ Furthermore, FERMT2 exerts a pro-cancer effect by promoting cytokine secretion in PSCs.^24^ In this study, analysis of pancreatic cancer single-cell sequencing data and FERMT2 IHC staining of tissue protein microarrays demonstrated that FERMT2 was predominantly expressed in pancreatic CAFs in pancreatic cancer tissues. In addition, the FERMT2 IHC scores were positively correlated with clinical staging, indicating the potential clinical relevance of FERMT2. GSEA revealed a positive correlation between FERMT2 expression and the proliferation and migration of fibroblasts. Functional experiments further confirmed that miR-138-5p regulated the proliferation and migration of CAFs by modulating the FERMT2 expression. The co-IP assay results demonstrated a direct interaction between FERMT2 and TGFBR1 in CAFs. Western blotting analysis revealed that miR-138-5p suppressed the expression of TGFBR1 through FERMT2, suppressing the activation of SMAD2/3. It is commonly acknowledged that the TGF-β/Smad signaling pathway serves an essential function in tumor fibrosis.^25^ The inactivation of the TGF-β/Smad signaling pathway in CAFs can lead to the suppression of CAF activation and pro-fibrotic factor secretion, the inhibition of CAF-mediated ECM remodeling, the downregulation of CAF-induced tumor cell invasion and metastasis, and the reversal of the pro-tumorigenic effects exerted by CAFs on therapy resistance. In addition, the co-IP assay showed that FERMT2 and PYCR1 interact directly in CAFs. Previous studies indicated that FERMT2 forms a complex with PYCR1 in human lung fibroblasts. The loss of FERMT2 suppressed the expression of PYCR1, impaired proline synthesis, and attenuated fibroblast activation.^26^ Furthermore, PYCR1-mediated proline synthesis is a major regulatory factor for collagen production in breast CAFs. In the breast cancer cell/breast CAF co-transplantation models, PYCR1 downregulation in CAFs can decrease tumor collagen content and effectively suppress tumor growth and metastasis. Herein, we discovered that FERMT2 regulates the PYCR1 expression in pancreatic CAFs, modulating proline synthesis and collagen production. The co-loading of miR-138-5p and the anti-fibrotic drug PFD into the EVs is a novel strategy. Incorporating miR-138-5p into engineered EVs was made possible by its downregulation in pancreatic CAFs. This combination strategy utilizes the anti-fibrotic activity of miR-138-5p and the anti-fibrotic action of PFD. Thus, miR-138-5p and PFD exert synergistic effects on CAFs to regulate fibrosis. The downstream effects of engineered EVs on the TGF-β/Smad signaling pathway further demonstrated their intricate regulatory mechanisms. The engineered EVs disrupt the CAF-mediated pro-fibrotic processes by inhibiting TGF-β signaling. This provides a potential avenue for reversing the aggressive tumor-promoting effects associated with the fibrotic microenvironment.

In vitro experiments suggested that engineered IEVs-PFD/138 exerted inhibitory effects on the proliferation and migration of pancreatic CAFs. IEVs-PFD/138 effectively suppressed the TGFBR1-mediated activation of the TGF-β signaling pathway by modulating FERMT2. In addition, IEVs-PFD/138 inhibited the PYCR1-mediated synthesis of proline, regulating collagen biosynthesis. Furthermore, IEVs-PFD/138 significantly downregulated the secretion of TGFB1, IL6, and CXCL12 by CAFs. These findings reveal the multifaceted mechanisms through which IEVs-PFD/138 treatment exerts inhibitory effects on the activation and fibrotic processes of pancreatic CAFs. IEVs-PFD/138 effectively suppressed the pro-cancer effects of CAFs. In a 3D multicellular tumor spheroid model, IEVs-PFD/138 enhanced the permeability of the tumor spheres. This increased permeability could facilitate the penetration of GEM, improving its therapeutic efficacy. In the xenograft mouse model, treatment with IEVs-PFD/138 suppressed tumor growth and modulated CAF activation in the TME. These findings suggest that treatment with IEVs-PFD/138 exerts inhibitory effects on tumor growth and promotes the reprogramming of CAFs in the TME. The combination of IEVs-PFD/138 and GEM exerted potent anti-tumor effects in the PDX model through the modulation of CAF activation and the downregulation of ECM components and tumor pressure. This resulted in improved intra-tumoral perfusion of GEM and increased sensitivity of tumor cells to GEM. IEVs-PFD/138 improved the therapeutic efficacy of GEM and suppressed metastasis in an orthotopic model of desmoplastic pancreatic cancer. The enhanced inhibitory properties of GEM on tumor growth and metastasis can be attributed to the modulation of pancreatic CAFs by IEVs-PFD/138. IEVs-PFD/138 counteracted the tumor-promoting effects of CAFs by modulating their activation, which resulted in TME remodeling. Subsequently, this remodeling suppressed tumor cell proliferation and invasion.

## Conclusions

This study demonstrated a novel and promising approach for treating desmoplastic pancreatic cancer using engineered EVs (IEVs-PFD/138) that can effectively target CAFs in the TME. miR-138-5p and PFD, which were used for the dual inhibition of CAFs, decreased fibrosis in the TME. In addition to enhancing the penetration of therapeutic agents, this approach directly suppressed the tumor-promoting effects of CAFs. The combination of IEVs-138/PFD with the standard chemotherapy drug GEM showed encouraging results, displaying a synergistic effect that significantly curbed tumor growth and metastasis in mouse models. These findings suggest that the application of engineered EVs is a promising therapeutic strategy for fibrosis-related cancers and offers novel insights for tackling the dense stroma characteristics of these malignancies. This targeted approach can enhance the efficacy of existing chemotherapy regimens, contributing to improved outcomes and enhanced quality of life for patients with desmoplastic pancreatic cancer.

## Materials and methods

### Cell lines and animals

The outgrowth method was employed to extract primary CAFs and NFs from pancreatic cancer tissues or para-cancer tissues. Fresh tissues were obtained from patients undergoing pancreatic cancer surgery and sectioned into about 3 mm in diameter. The tissues were removed when the fibroblasts migrated out. Dulbecco’s modified Eagle medium (DMEM)/F12 (Pricella Life Science&Technology Co.,Ltd) mixed with 100 U/mL penicillin, 100 μg/mL streptomycin (BioChannel Biological Technology CO., Ltd), and 10% fetal bovine serum (FBS) (Pricella Life Science&Technology Co.,Ltd) was used to culture the fibroblasts. CAFs and NFs were identified by analyzing the fibroblasts markers ACTA2, FAP, and FSP. BMSCs were purchased from Cyagen Biosciences and cultured in DMEM/F12 containing 100 U/mL penicillin, 100 μg/mL streptomycin, and 10% FBS. The identification of BMSCs was based on their differentiation ability and the analysis of surface markers by flow cytometry. The human pancreatic cancer cell line (PANC-1 cells) was sourced from Pricella Life Science&Technology Co.,Ltd and maintained in DMEM containing 100 U/mL penicillin, 100 μg/mL streptomycin, and 10% FBS. A temperature of 37 °C and a CO_2_ concentration of 5% were used for all cell cultures. The cells were passaged using 0.25% trypsin-EDTA (BioChannel Biological Technology CO., Ltd) and cryopreserved at −80 °C using serum-free cell freezing medium (New Cell & Molecular Biotech).

BALB/c nude mice (male, 4–6 weeks old) and C57/B6 mice (male, 4–6 weeks old) were purchased from Gempharmatech Co., Ltd, C-NKG mice (male, 4–6 weeks old) were purchased from Cyagen Biosciences. All mice were raised in SPF-grade environments.

Animal experiments were approved by the Animal Ethics Committee of Southeast University. Experiments involving human tissues were approved by the clinical ethics committee of the Affiliated Hospital of Nantong University and performed according to the guidelines of the Declaration of Helsinki.

### Immortalization of human pancreatic CAFs and BMSCs

Human patient-derived CAFs (passage 3) and primary BMSCs were immortalized by infection with human telomerase reverse transcriptase (TERT) lentivirus (Genechem), following a methodology referenced from previous studies.^27,28^ Cells were maintained in puromycin and the immortalized CAFs and BMSCs were selected.

### FISH assay of miR-138-5p

The presence of a specific miR-138-5p within CAFs or NFs was visualized via FISH. Cells were cultured on 48-well slides, followed by 4% paraformaldehyde (PFA) fixation, and Triton X-100 permeabilization. Next, a miR-138-5p-CY3 probe (Generay) was added to the cells for incubation to allow the hybridization of the probe with the target sequences overnight at 37 °C. After washing to remove unbound probes, DAPI was utilized for cell nuclei staining. A confocal microscope was employed to capture the images.

### Cell transfection

Six-well plates were inoculated with CAFs, which were maintained in culture till 70% confluency. This was followed by co-transfecting the cells with 100 pmol NC-mimic and miR-138-5p mimic (GenePharma), 200 pmol NC-inhibitor and miR-138-5p inhibitor (GenePharma), or 100 pmol si-FERMT2 (RioBio) and 2 µg plasmid expressing PYCR1 (GenePharma) using 4 μL Lipo8000 (Beyotime).

### Construction of engineered EVs

Primary BMSCs were cultured and infected with hTERT-encoding lentivirus (Genechem) to establish immortalized BMSCs. The culture media of immortalized BMSCs was collected, and cells and dead cells were removed by centrifuging the sample at 300 × *g* for 10 min and at 2000 × *g* for another 10 min. Next, the cell debris was eliminated by centrifuging the media at 10,000 × *g* for half an hour. Further, the media underwent ultracentrifugation using an ultracentrifuge (Beckman) at 200,000 × *g* for 70 min. A temperature of 4 °C was maintained for all the centrifugation steps. EVs were re-suspended in sterile PBS before being preserved at −80 °C for later use. The EVs were loaded with PFD (MCE) and miR-138 mimics using sonication. To load PFD and miR-138-5p mimics into the EVs, 100 µL EVs (1 mg/mL) were incubated with 100 µL PFD (20 mg/mL) and 1OD miR-138-5p mimics at 37 °C for 30 min and sonicated. The sonication conditions were as follows: 60 W, 6 cycles of 3 s pulse, and 10 s pause, each cycle at 4 °C, followed by a 10 min cooling period on ice. Thereafter, the exosomes were subjected to incubation for 1 h at 37 °C for the stabilization of the EV membrane. In addition, DSPE-PEG-CRYYRITY (RYYRITY peptide had integrin α5-targeting ability) was synthesized, and 100 μg of EVs-PFD/138 was incubated with 50 μg of DSPE-PEG-CRYYRITY at 37 °C for 1 h. Subsequently, the resultant product was re-suspended in PBS and subjected to ultracentrifugation to obtain engineered EVs (IEVs-PFD/138). The loading efficiency of PFD in IEVs-PFD/138 was determined using UV absorbance spectroscopy. The relative miR-138-5p expression level was determined using qRT-PCR analysis.

### Identification of EVs

TEM was utilized to analyze the EVs’ morphology. The expression levels of specific marker proteins associated with EVs were examined using western blotting analysis. NTA was employed to ascertain the EVs’ size distribution. The EVs were immobilized on copper grids by placing a drop of EV suspension onto the grid and allowing it to be adsorbed for several minutes. Excess liquid was gently removed using filter paper. Next, phosphotungstic acid was used to stain the EVs on the grid. A filter paper was used to remove any excess stains. Then, the stained EVs on the grid were imaged under a transmission electron microscope (TEM). In addition, the presence of specific markers, namely, CD9, CD63, Alix, and TSG101, on the surface of EVs and IEVs-PFD/138 was determined. The particle size distribution of EVs and IEVs-PFD/138 was determined using NTA.

### In vitro cellular internalization of EVs

Confocal microscopy and flow cytometry were applied to study the uptake of EVs by CAFs. CAFs were seeded in 24-well plates (20 μg) for confocal microscopy or 6-well plates (100 μg) for flow cytometry. EVs were labeled with DID (US, BRIGHT, Suzhou, China) or loaded with FAM-miR-138-5p. The cells were incubated with DID-labeled EVs or FAM-miR-138-5p-loaded EVs for 8 h. Finally, PBS was used to wash the samples to isolate the non-internalized EVs. Staining the cells in the 24-well plate with DAPI allowed for the visualization of the cell nuclei. The fluorescence signals of DID and FAM were captured with a confocal laser scanning microscope. Subsequently, the cells in the 6-well plate were dissociated into single-cell suspension using trypsin-ethylenediaminetetraacetic acid. A flow cytometer with the DID and FAM channels was employed to measure the fluorescence intensity. In addition, miR-138-5p expression in CAFs treated with EVs or IEVs was measured via qRT-PCR analysis.

### Biodistribution of EVs in vivo

A fibrotic pancreatic cancer mouse model was designed by co-transplanting PANC-1 cells and CAFs to study the impact of IEVs-PFD/138 on CAFs in pancreatic cancer. Each nude mouse was injected with 4 × 10^6^ PANC-1 cells and 1 × 10^6^ CAFs into the subcutaneous flank region. As soon as the tumor volume reached 500 mm^3^ (*n* = 3), intravenous injections of EVs or IEVs at a dosage of 100 μg/mouse were administered. At 2, 4, 8, and 12 h following injection, the in vivo fluorescence images were captured utilizing an IVIS. For ex vivo immunofluorescent staining, The tissues from the tumors were removed 4 h after injection and then fixed in 4% PFA for another 24 h. Next, Two solutions containing 20 and 30% sucrose were used to dehydrate the tissues for 24 h each. Afterward, Following embedding in O.C.T., the tumor tissues were cut into 15-μm-thick sections. DAPI staining was performed on the sections to visually differentiate the cell nuclei. The fluorescence signals of DID were captured using fluorescence confocal microscopy.

### RNA isolation and qRT-PCR analysis

Six-well plates were inoculated with CAFs followed by 24 h or transfection. RNA was extracted utilizing a TRIZOL reagent, as directed by the manufacturer. Then, the concentration of RNA was measured. The extracted RNA (1000 ng) was reverse-transcribed into complementary DNA (cDNA) utilizing the SweScript RT I First Strand cDNA synthesis kit (Servicebio). cDNA was diluted in the ratio of 1:5. qRT-PCR analysis was implemented utilizing 2× SYBR Green qPCR Master Mix (Servicebio) and ThermoFisher StepOne^TM^. miR-138-5p expression was determined with miR-138-5p Bulge-Loop^TM^ RT Primer, Bulge-Loop^TM^ Forward Primer, and Bulge-Loop^TM^ Reverse Primer (Ribobio). The internal control was U6. The 2^−ΔΔCt^ method was applied to calculate the gene expression levels.

### Western blotting analysis

A radioimmunoprecipitation assay lysis buffer (RIPA) (Solarbio) supplemented with phosphatase and protease inhibitors was used to lyse the cells. This was followed by centrifuging the lysates at 12,000 × *g* and 4 °C for 20 min. Thereafter, electrophoresis on a sodium dodecyl sulfate-polyacrylamide gel was performed on the supernatant before loading the resolved proteins onto a polyvinylidene fluoride membrane. Subsequently, NcmBlot blocking buffer was used to block the membrane for 15 min at room temperature, followed by overnight incubation with primary antibodies at 4 °C. Afterward, PBS containing Tween-20 (PBST) was used to rinse the membrane thrice after which it was treated with the appropriate secondary antibody. The chemiluminescence detection reagent (Yeasen) was employed to visualize the immunoreactive signals.

### Dual-luciferase reporter gene assay

miRTarBase, miRDB, TarBase, miRmap, miRDIP, and MicroT were utilized to predict possible miR-138-5p target genes. The miR-138-5p-FERMT2 binding sites were predicted. Synthesis of wild-type and mutant sequences of FERMT2 was performed, before cloning them into the pmiR-GLO luciferase vector (Promega, Madison, WI, USA). The plasmids and either a miR-138-5p mimic or an NC-mimic were co-transfected into HEK-293T cells using lipo8000 for 48 h. The dual-luciferase reporter assay kit (Genepharma) was utilized to measure the activities of both Renilla and luciferase.

### Co-IP assay

Ten-cm plates were used to seed the cells, which were then grown to 90% confluency. Next, 500 μL of NP-40 lysis buffer mixed with a protease inhibitor cocktail (Beyotime) was used to lyse the cells for half an hour at 4 °C. Protein A/G magnetic beads (MCE) were pre-cleared with PBST and subjected to incubation for an hour with antibodies at room temperature. IP was performed by incubating cell lysates with mouse IgG (Servicebio), rabbit IgG (CST), rabbit anti-FERMT2 (Proteintech), rabbit anti-PYCR1 (Proteintech), or mouse anti-TGFBR1 antibodies (Santa Cruz) for an entire night at 4 °C. Thereafter, the samples were subjected to western blotting using rabbit anti-FERMT2 (Proteintech), rabbit anti-PYCR1 (Proteintech), or rabbit anti-TGFBR1 (Servicebio) antibodies.

### EdU, CCK-8, would-healing, and transwell assays

The proliferative rate of CAFs and pancreatic cancer cells was determined using the EdU assay and CCK-8 assay. Cells were incubated with EdU, which will be incorporated into replicating DNA during the cell cycle’s S phase. Next, the cells were fixed, permeabilized, and incubated with Azide Alexa Fluor 594 to detect the incorporated EdU. The cells were imaged, and the percentage of proliferating cells was computed. For CCK-8 assay, cells were treated with a 10% CCK-8 solution (APExBIO), followed by an incubation at 37 °C in a cell culture incubator for 2 h. Absorbance values were measured using a microplate reader at a wavelength of 450 nm. The migration of CAFs and pancreatic cells was evaluated using the wound-healing assay. Six-well plates were used to seed the cells, which were then grown to confluence. Next, a sterile pipette tip was used to introduce a controlled wound into the monolayer. A serum-free basic medium was used to replace the culture medium, after which the cells were allowed to migrate toward the wound area for 24 h. The migration of cells into the wound area was documented using microscopy at 0 h (initial time point) and 24 h (final time point). The invasive potential of pancreatic cancer cells was examined using the transwell assay. Cell suspension (100 μL) without FBS was placed in an 8-μm transwell upper chamber. There were 600 μL of medium with 10% FBS in the bottom chamber. Following a 12-h incubation period, 4% PFA was used to fix the invading cells, and crystal violet was used for staining them. The images were captured under a microscope.

### ELISA

After obtaining the cell culture supernatants, cellular debris was eliminated by centrifuging the sample at 300 × *g* for 10 min. The expression levels of IL6, TGFB1, and CXCL12 were quantified using the human IL-6 ELISA kit, human TGF-β1 ELISA kit, and human CXCL12/SDF-1 ELISA kit, respectively, purchased from MULTI SCIENCES, following the directions stipulated by the manufacturer. Briefly, the samples (100 μL) were introduced into the designated wells of the ELISA plate coated with specific antibodies and incubated with 50 μL of detection antibodies at room temperature while being agitated at 150 rpm for 2 h. Once the samples had been washed, they were subjected to incubation with 100 μL of diluted horseradish peroxidase-conjugated streptavidin at room temperature with shaking at 150 rpm for 45 min. After additional washing, each well received 100 μl of TMB substrate, which was subjected to incubation at room temperature for 5–30 min in darkness. Ultimately, 100 μL of stop solution was added to the samples and incubated. A microplate reader was used to measure the absorbance of the reaction mixture at 450 and 630 nm. The IL6, TGFB1, and CXCL12 expression levels were determined by comparing the absorbance values of the samples to those of the standard analytes from a standard curve generated using specified concentrations of the analytes.

### Ex vivo explant culture

We adopted the ex vivo explant culture approach as described in a previous study.^27^ Before collecting tumor tissue, hemostatic gelatin sponges were immersed in a culture medium comprising high-glucose DMEM with 10% FBS and 1× antibiotic/antimycotic solution. Following saturation, the sponges were transferred to 24-well plates, and each well was filled with 500 μL of culture solution, which completely covered the sponge’s bottom half. The plates were then preserved in a 37 °C/5% CO_2_ incubator until needed. Tumor tissue was cut into 2 mm diameter sections and embedded into the sponges. The culture medium was replaced daily.

### In vivo anti-tumor effect of IEVs-PFD/138 on subcutaneous xenograft mouse model

To establish a subcutaneous stroma-rich pancreatic cancer model, BALB/c nude mice were injected with a mixture of PANC-1 cells (4 × 10^6^ cells) and CAFs (1 × 10^6^ cells) into their right flank. Upon the tumor volume reaching 50 mm^3^, the mice were classified into four distinct groups at random (*n* = 5/group): PBS-treated, IEVs-PFD-treated, IEVs-138-treated, and IEVs-PFD/138-treated groups. For 21 days, the mice received intravenous injections of PBS or different EVs one time every three days. The tumor dimensions were recorded once every 3 days until day 21. The formula for the tumor volume (*V*) was: *V* = (length × width^2^)/2. On day 21, the tumors and major organs were harvested from the mice. The organs were subjected to HE staining. Meanwhile, the tumors were subjected to Masson staining, KI67, ACTA2, FAP, FSP, and PYCR1 immunofluorescence staining, and western blotting analysis to analyze the FERMT2, PYCR1, TGFBR1, and p-SMAD2/3 levels. In addition, another group of 5 mice from each group received the same treatment to examine the survival duration (death was defined as the time point when the tumor volume reached 1000 mm^3^).

### In vivo anti-tumor effect of IEVs-PFD/138 on pancreatic cancer patient-derived subcutaneous xenograft (PDX) mouse model

Before establishing the PDX model, informed consent was obtained from the patients after providing them with comprehensive information. The Ethics Committee of Nantong University Affiliated Hospital approved the study protocol. Fresh pancreatic cancer tissue samples were aseptically dissected into fragments measuring approximately 2–3 mm in diameter. The C-NKG mice’s right flank was then implanted with these pieces. After two passages in vivo, the third-generation (F3) PDX models were used for subsequent experiments. Upon attaining a tumor volume of 50 mm^3^, three randomized groups of mice were established (*n* = 5): the PBS group, the gemcitabine group, and the IEVs-PFD/138+gemcitabine group. Different treatments were administered via tail vein injection every 3 days for a total of 8 treatments. Tumor dimensions were recorded every 3 days until day 24. On day 24, the tumors were harvested, and their weights were recorded. In addition, tumor pressure was measured according to a previously published method^29^, where the tumor was cut along 80% of its length, soaked in HBSS solution for 10 min, and the tumor’s opening size and short diameter were determined using calipers to calculate the tumor pressure. Histological examination including HE staining, Masson staining, immunohistochemistry for ACTA2 and KI67, and TUNEL staining was performed on the tumor tissues. Furthermore, FERRMT2, PYCR1, TGFBR1, P-smad2/3, HIF1a, and ENT1 protein expression levels in the tumor tissues were determined by Western blotting analysis. In addition, another group of 5 C-NKG mice in each treatment group was included to observe their survival time, with a tumor volume of 1000 mm^3^ considered as the endpoint for survival analysis.

### In vivo anti-tumor effect of IEVs-PFD/138 on orthotopic xenograft immunodeficiency mouse model

BALB/c nude mice were injected with a combination of PANC-1-luc cells (2 × 10^6^) and CAFs (0.5 × 10^6^) into their pancreas tail to establish an orthotopic stroma-rich pancreatic cancer model. Three groups of mice were established at random after 14 days of tumor growth (*n* = 5): the PBS group, the gemcitabine group, and the IEVs-PFD/138+gemcitabine group. Different treatments were administered via tail vein injection every 3 days for a total of 7 treatments. At days 0, 7, 14, and 21, D-luciferin potassium (3 mg/mouse) was intraperitoneally injected for bioluminescent imaging. On day 21, the mice were euthanized, and the presence of abdominal metastases and the volume of ascites were assessed. The pancreas was harvested, and the dimensions of the orthotopic tumor were measured. In addition, the tumor tissues were subjected to Masson staining, TUNEL staining, and immunofluorescence staining for VIM and N-cad. The number of liver metastatic nodules was counted, and HE staining was conducted on the liver to count the microscopic liver metastatic nodules.

### In vivo anti-tumor effect of IEVs-PFD/138 on orthotopic xenograft immunocompetent mouse model

Due to the presence of immune cells in the tumor microenvironment, we established an orthotopic pancreatic cancer model in C57/B6 mice to observe the therapeutic effects of IEVs-PFD/138 in immunocompetent mice. Initially, C57/B6 mice were injected with a mixture of Panc02-luc cells (2 × 10^6^) and NIH-3T3 cells (0.5 × 10^6^) into the tail of the pancreas. After 7 days of tumor growth, three groups (*n* = 5) of mice were created at random: the PBS group, the gemcitabine group, and the IEVs-PFD/138+gemcitabine group. Various treatments were administered via tail vein injection for a total of 4 treatments. At days 0, 6, and 12, D-luciferin potassium (3 mg/mouse) was intraperitoneally injected for bioluminescent imaging. The pancreas was harvested, and the dimensions of the orthotopic tumor were measured. The FERRMT2, PYCR1, TGFBR1, P-smad2/3, and ENT1 protein expression in the tumor tissues were determined by Western blotting analysis. Histological examination, including Masson staining, TUNEL, immunofluorescence for KI67, ACTA2, and collagen1 staining, was performed on the tumor tissues.

### Immunohistochemistry and immunofluorescence

The tissue was fixed with 4% paraformaldehyde embedded in paraffin, dewaxed with xylene, and dehydrated with ethanol, and then repaired with 0.01 mM citrate buffer. The tissue section was incubated with rabbit anti-Ki67, rabbit anti-ACTA2, rabbit anti-FERMT2, rabbit anti-PYCR1, and rabbit anti-collagen1 antibodies at 4 °C overnight. HRP-conjugated secondary antibodies were added and allowed to incubate for 1 h at room temperature. Finally, slides were stained with DAB and counterstained with hematoxylin. Images were pictured by brightfield microscopy. For immunofluorescence, corresponding fluoresce-conjugated secondary antibodies were added and allowed to incubate for 1 h at room temperature. Finally, slides were stained with DAPI to visualize cell nuclei. Images were pictured by fluorescence confocal microscopy.

## Statistical analysis

The quantitative data were presented as means ± standard deviation (SD). Comparative analysis was performed with Student’s t-test (two-tailed unpaired) for two groups, and a one-way analysis of variance (ANOVA) test was used for >2 groups. GraphPad Prism 8.0 software (GraphPad Software, Inc., USA) was employed for all analysis of data. Differences with *p* < 0.05 were considered significant (*p < 0.05, **p < 0.01, and ***p < 0.001).

### Supplementary information

supplementary materials

original WB image

## Data Availability

All data generated or analyzed throughout this study have been incorporated into this published article along with its supplementary files. The Cancer Genome Atlas (TCGA) datasets referenced in the study are accessible through a public repository hosted on the cBioPortal website (https://www.cbioportal.org/). Public RNA-array datasets of CAFs and NFs analyzed in the study is available in Gene Expression Omnibus under accession no. GSE123377.
