# Supplementary material for: Engineered extracellular vesicles for targeted reprogramming of cancer-associated fibroblasts to potentiate therapy of pancreatic cancer
Source: Signal Transduct Target Ther. 2024 Jun 24;9:151. doi: 10.1038/s41392-024-01872-7 (PMC11194278; doi:10.1038/s41392-024-01872-7)
Supplement: Supplementary file 1 — supplementary materials [file 41392_2024_1872_MOESM1_ESM.docx]

Supplementary Materials for

Engineered extracellular vesicles for targeted reprogramming of cancer-associated fibroblasts to potentiate therapy of pancreatic Cancer

Pengcheng Zhou^1,2^, Xuanlong Du^2^, Weilu Jia^2^, Kun Feng^3^, Yewei Zhang^4^

Correspondence to: [zhangyewei@njmu.edu.cn](mailto:zhangyewei@njmu.edu.cn) (Yewei Zhang)

**This PDF file includes:**

Supplementary fig. 1 to Supplementary fig. 4


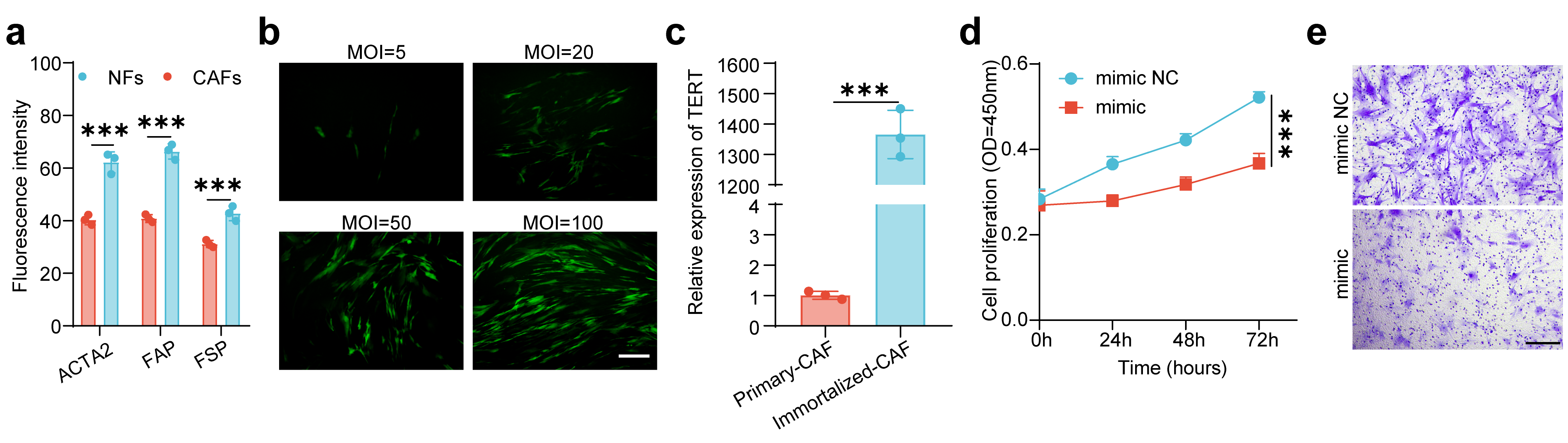


Supplementary fig. 1. Construction of immortalized cancer-associated fibroblasts (CAFs) and effects of miR-138-5p on CAFs (a) Fluorence intensity of ACTA2, FAP and FSP. Data represent the mean (± SD); n = 3 per group. (b) Human telomerase reverse transcriptase (TERT)-green fluorescent protein (GFP)-encoding lentivirus infection (green: GFP. scale bar = 100 μm). (c) Relative expression of *TERT* mRNA. Data represent the mean (± SD); n = 3 per group. (d) CCK8 of CAFs. Data represent the mean (± SD); n = 3 per group. (e)Transwell of CAFs (scale bar = 100 μm).


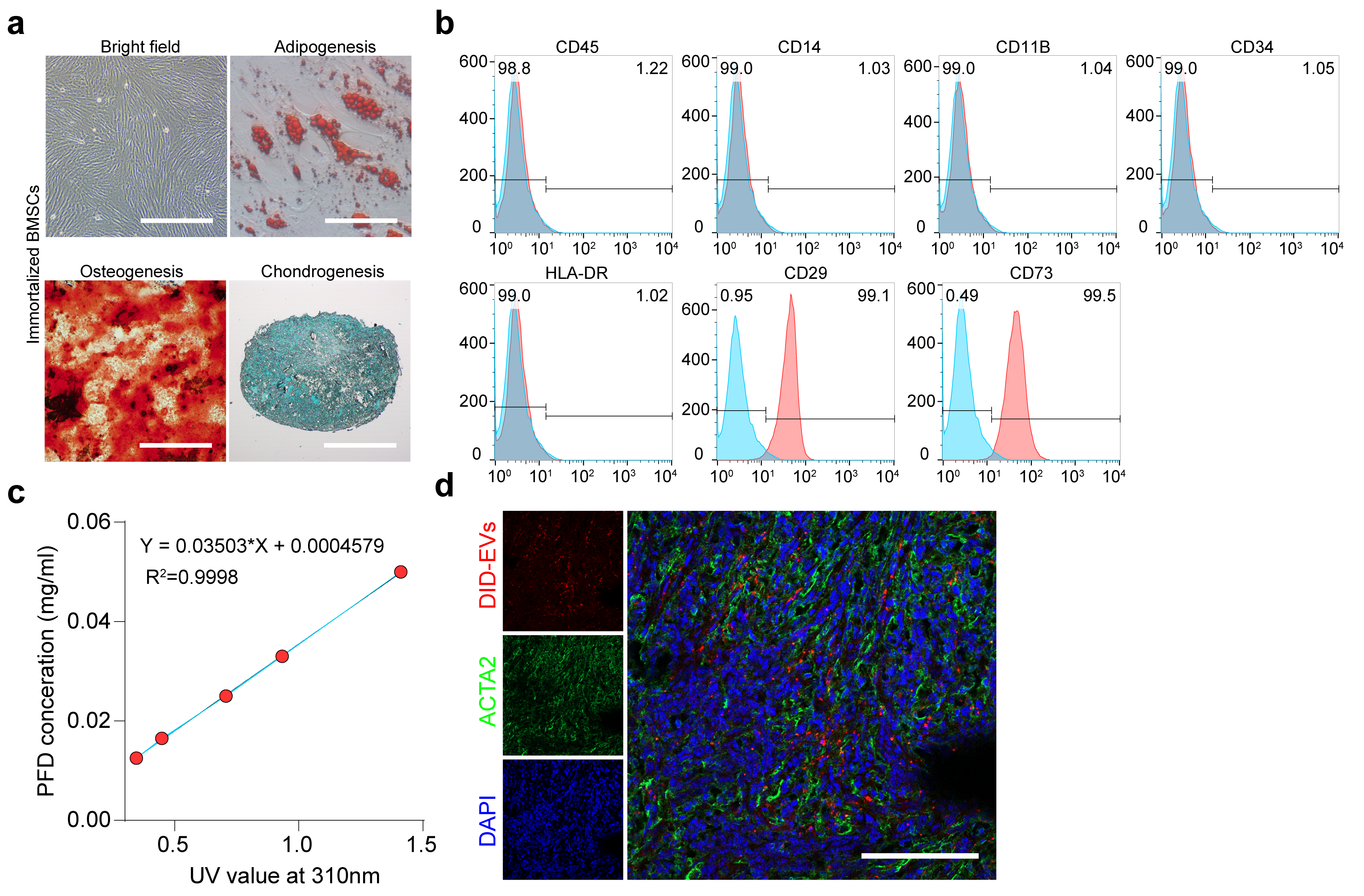
 **Supplementary fig. 2.** Identification of immortalized bone marrow mesenchymal stem cells (BMSCs). (a) Morphology and differentiation capacity of immortalized BMSCs (scale bar = 100 μm). (b) Identification of surface markers of immortalized BMSCs using flow cytometry. (c) PFD standard curve. (d) Fluorescent staining of ACTA2 and DID-EVs (Red: DID-EVs, green: ACTA2, blues: cell nuclei. cale bar = 100 μm).


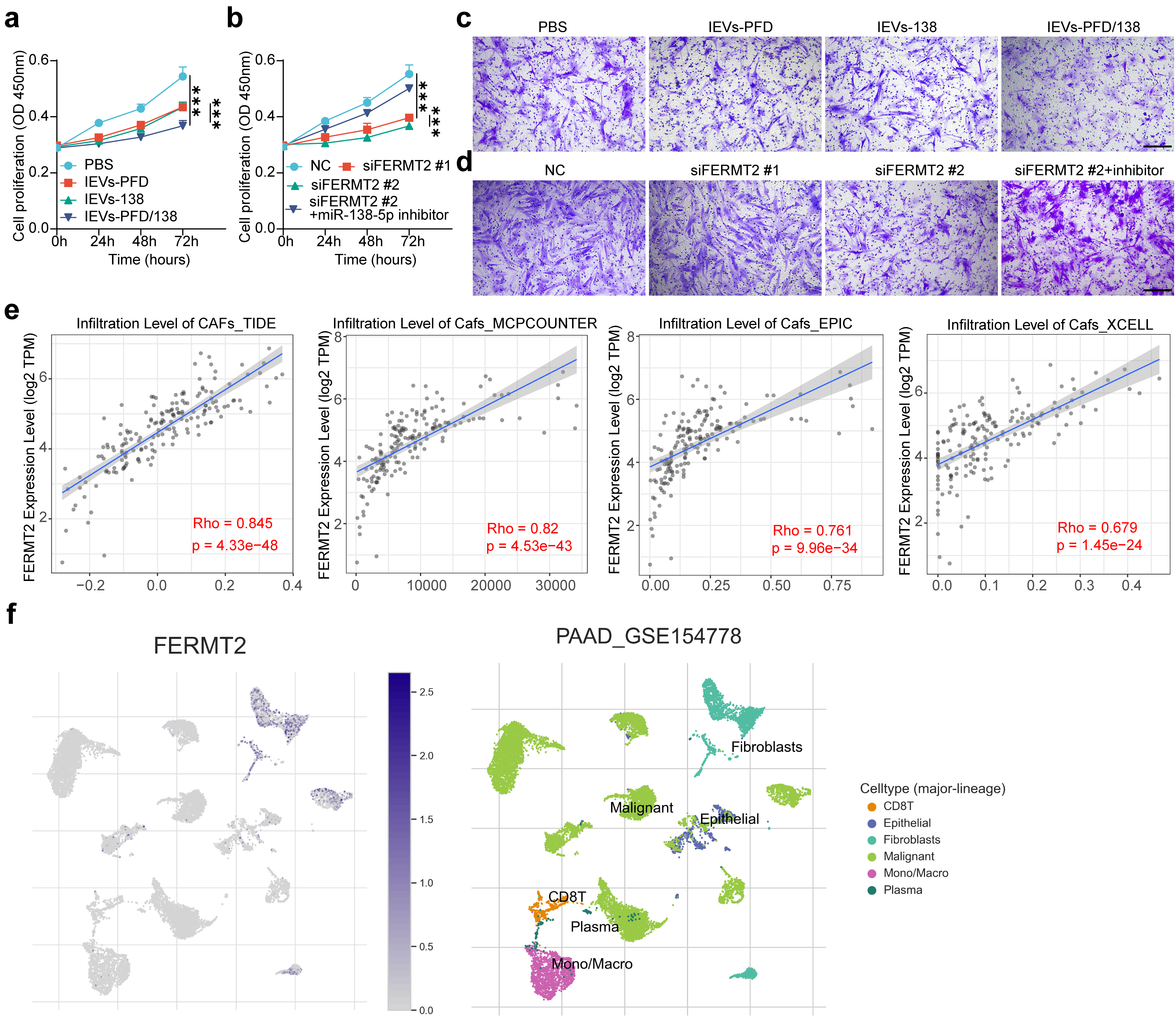
 Supplementary fig. 3. Functional experiments of cancer-associated fibroblasts (CAFs) and FERMT2 expression in CAFs. (a, b) CCK-8 of CAFs. Data represent the mean (± SD); n = 3 per group. (c, d) Transwell of CAFs. (e) FERMT2 expression was positively correlated with CAF infiltration levels according to TIMER database (http://timer.cistrome.org/). (f) FERMT2 was enriched in CAFs with the Tumor Immune Single-cell Hub (TISCH) database (http://tisch.comp-genomics.org/).


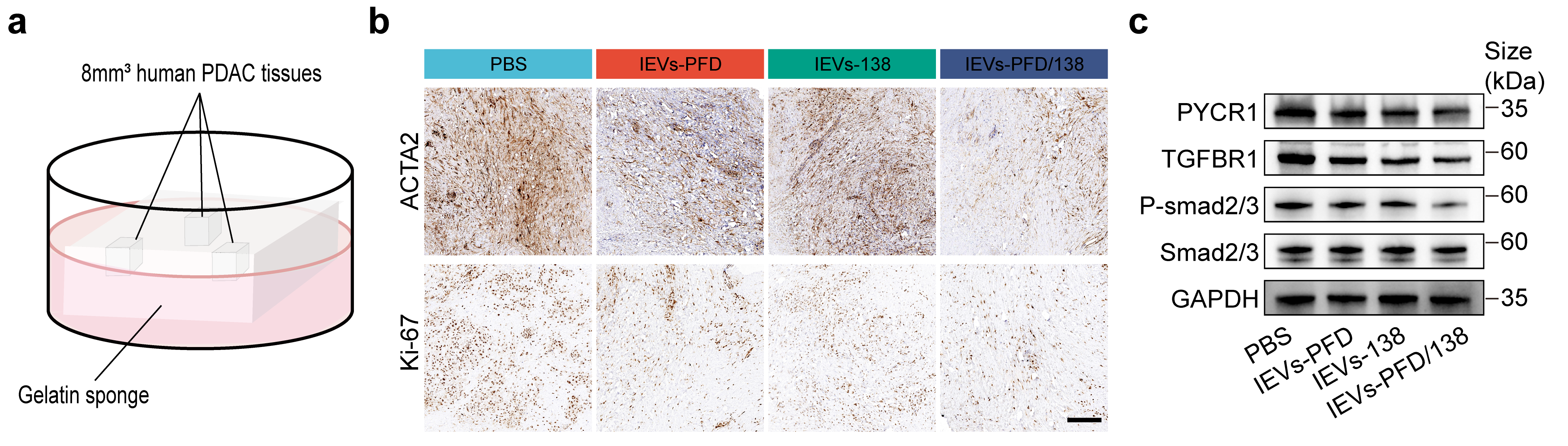
Supplementary fig. 4. 3D human tumor explants. (a) Schematic diagram. (b) ACTA2 and Ki67 immunohistochemical staining (scale bar = 200 μm). (c) Western blotting analysis of the PYCR1, PYCR1, TGFBR1, and P-SMAD2/3 expression levels in tumor explants subjected to different treatments.
