# Supplementary material for: Engineered extracellular vesicles for targeted reprogramming of cancer-associated fibroblasts to potentiate therapy of pancreatic cancer
Source: Signal Transduct Target Ther. 2024 Jun 24;9:151. doi: 10.1038/s41392-024-01872-7 (PMC11194278; doi:10.1038/s41392-024-01872-7)
Supplement: Supplementary file 2 — original WB image [file 41392_2024_1872_MOESM2_ESM.ppt]

## Slide 1
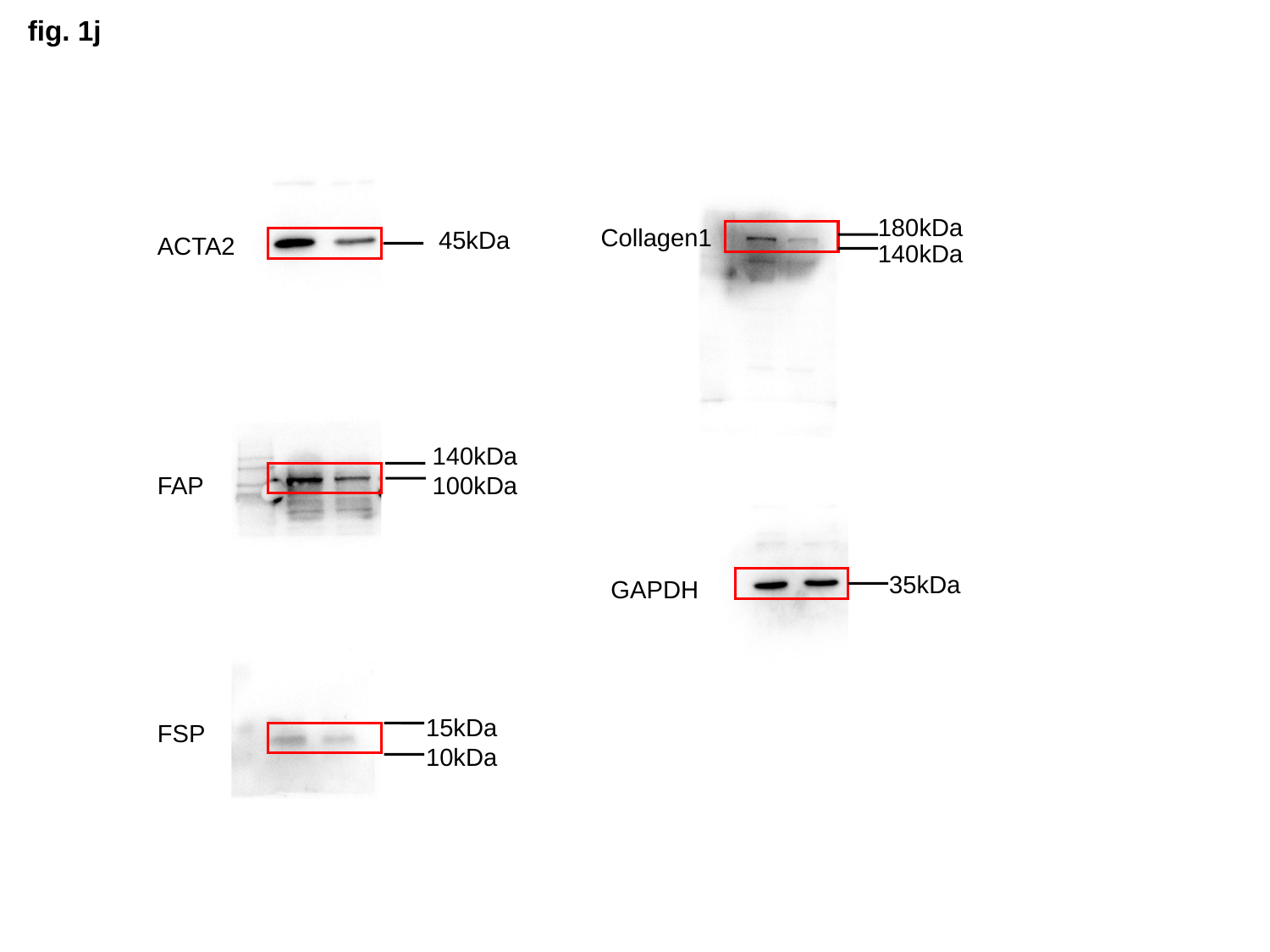

fig. 1j
180kDa
Collagen1
45kDa
ACTA2
140kDa
140kDa
FAP
100kDa
35kDa
GAPDH
15kDa
FSP
10kDa

## Slide 2
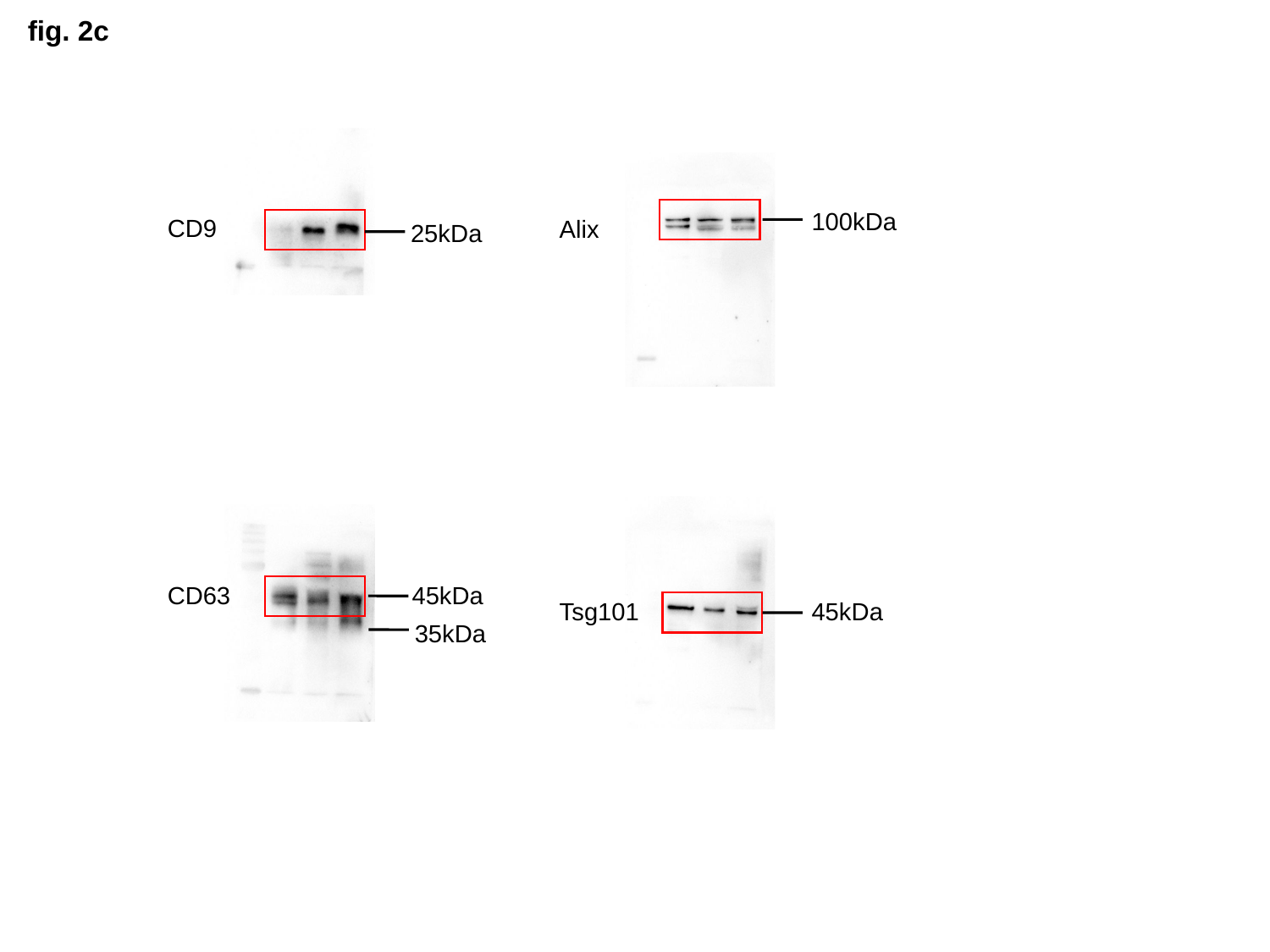

fig. 2c
100kDa
CD9
Alix
25kDa
CD63
45kDa
Tsg101
45kDa
35kDa

## Slide 3
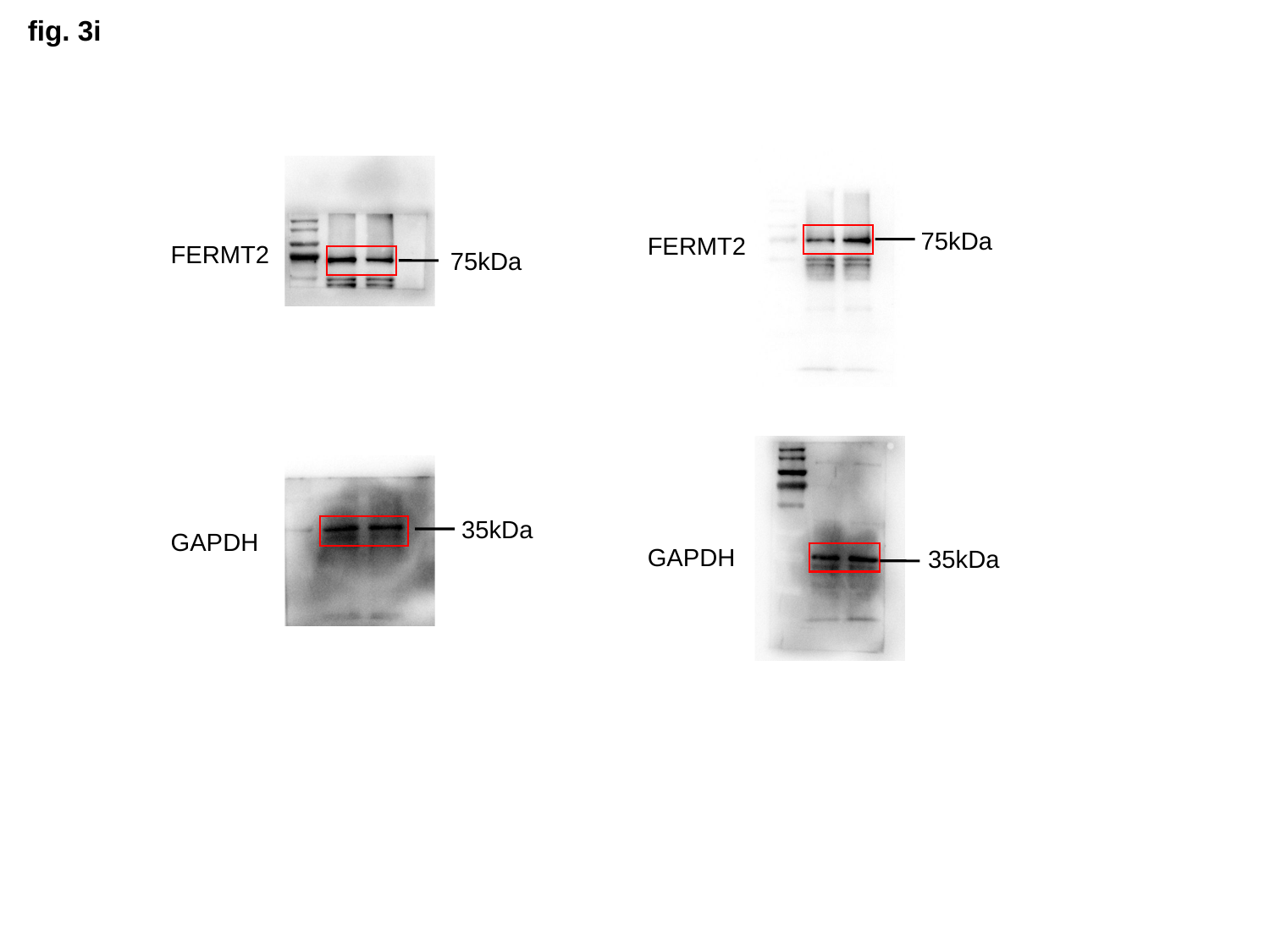

fig. 3i
75kDa
FERMT2
FERMT2
75kDa
35kDa
GAPDH
GAPDH
35kDa

## Slide 4
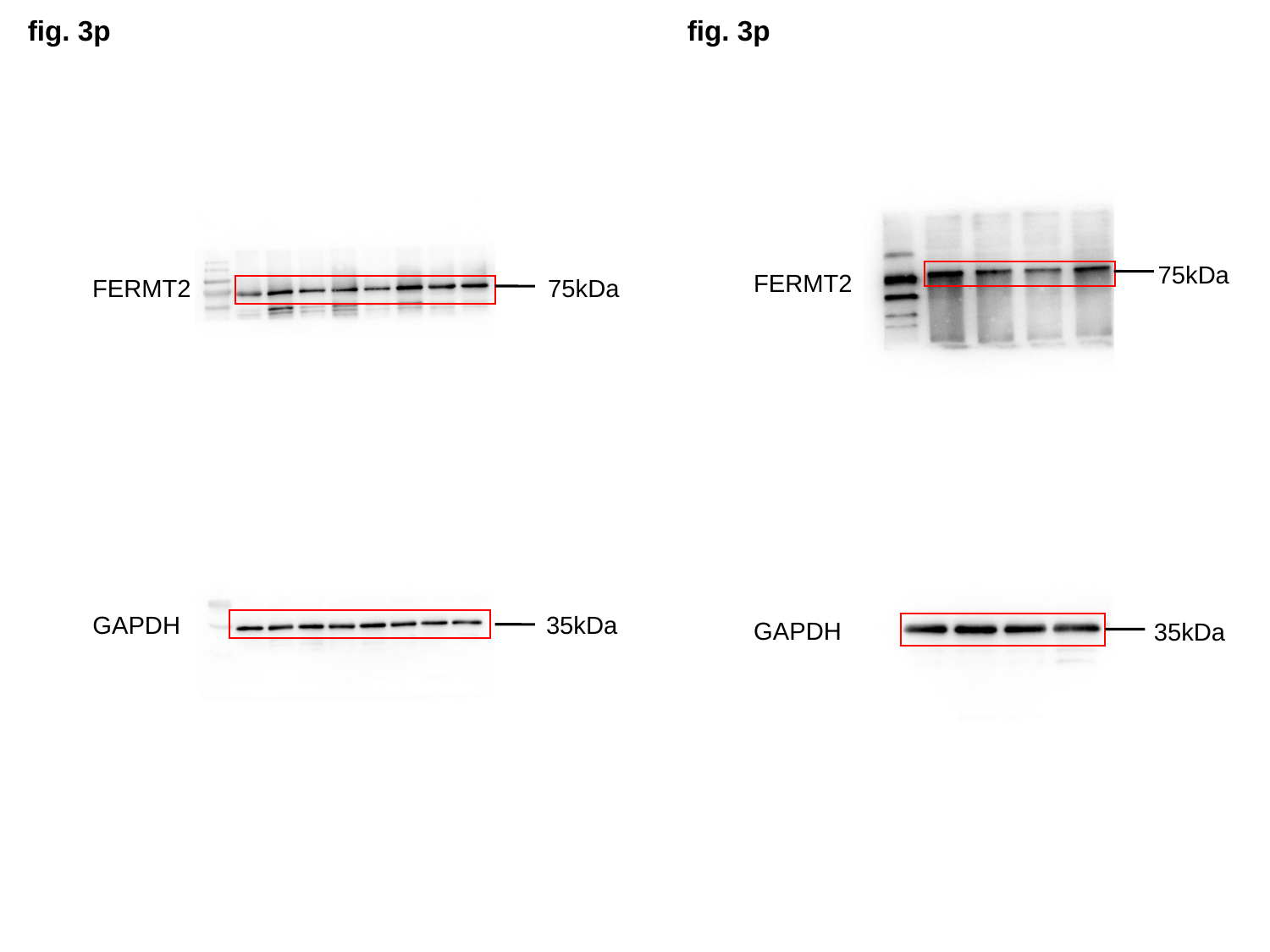

fig. 3p
fig. 3p
75kDa
FERMT2
FERMT2
75kDa
GAPDH
35kDa
GAPDH
35kDa

## Slide 5
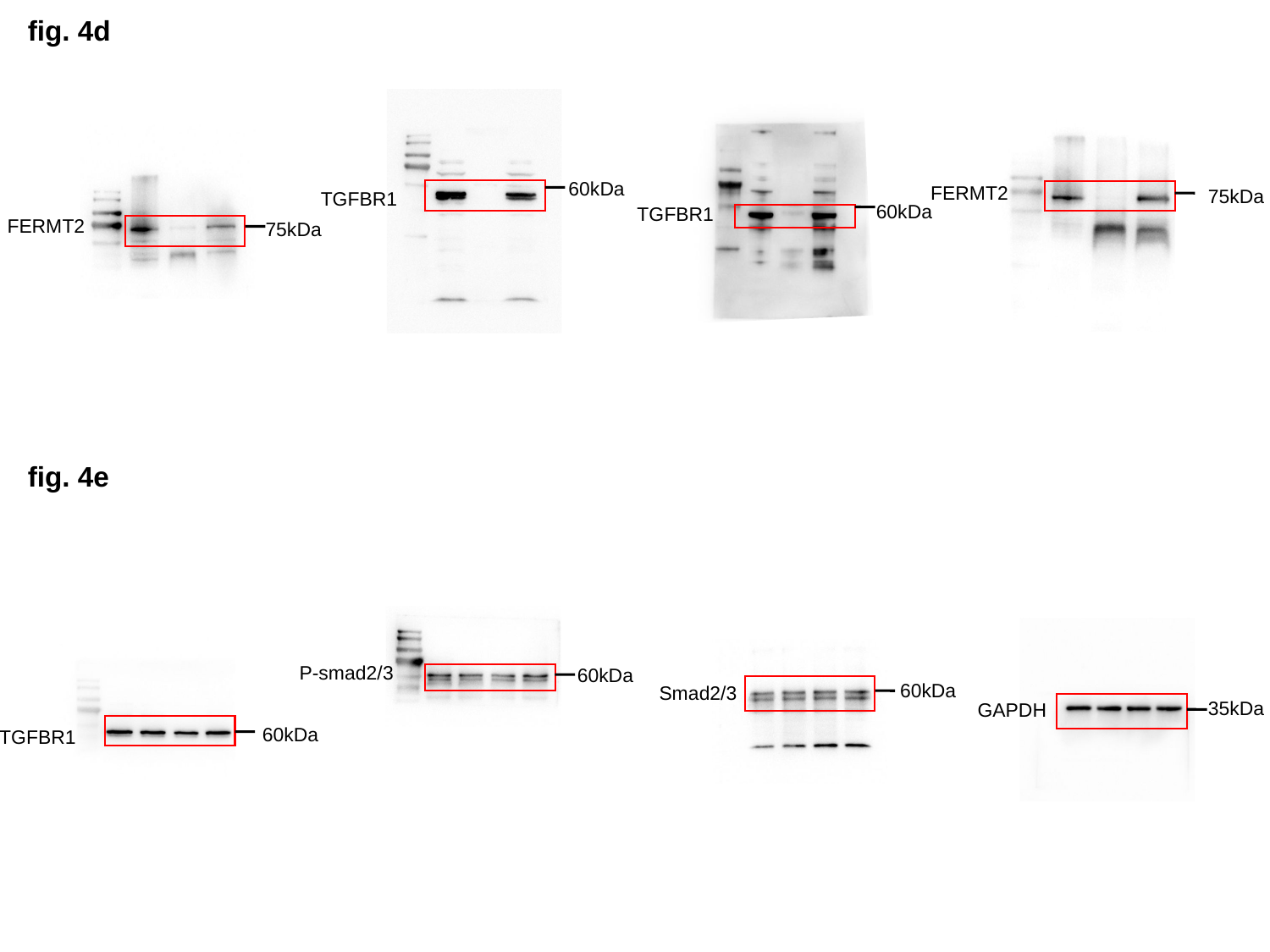

fig. 4d
60kDa
FERMT2
75kDa
TGFBR1
60kDa
TGFBR1
FERMT2
75kDa
fig. 4e
P-smad2/3
60kDa
60kDa
Smad2/3
35kDa
GAPDH
60kDa
TGFBR1

## Slide 6
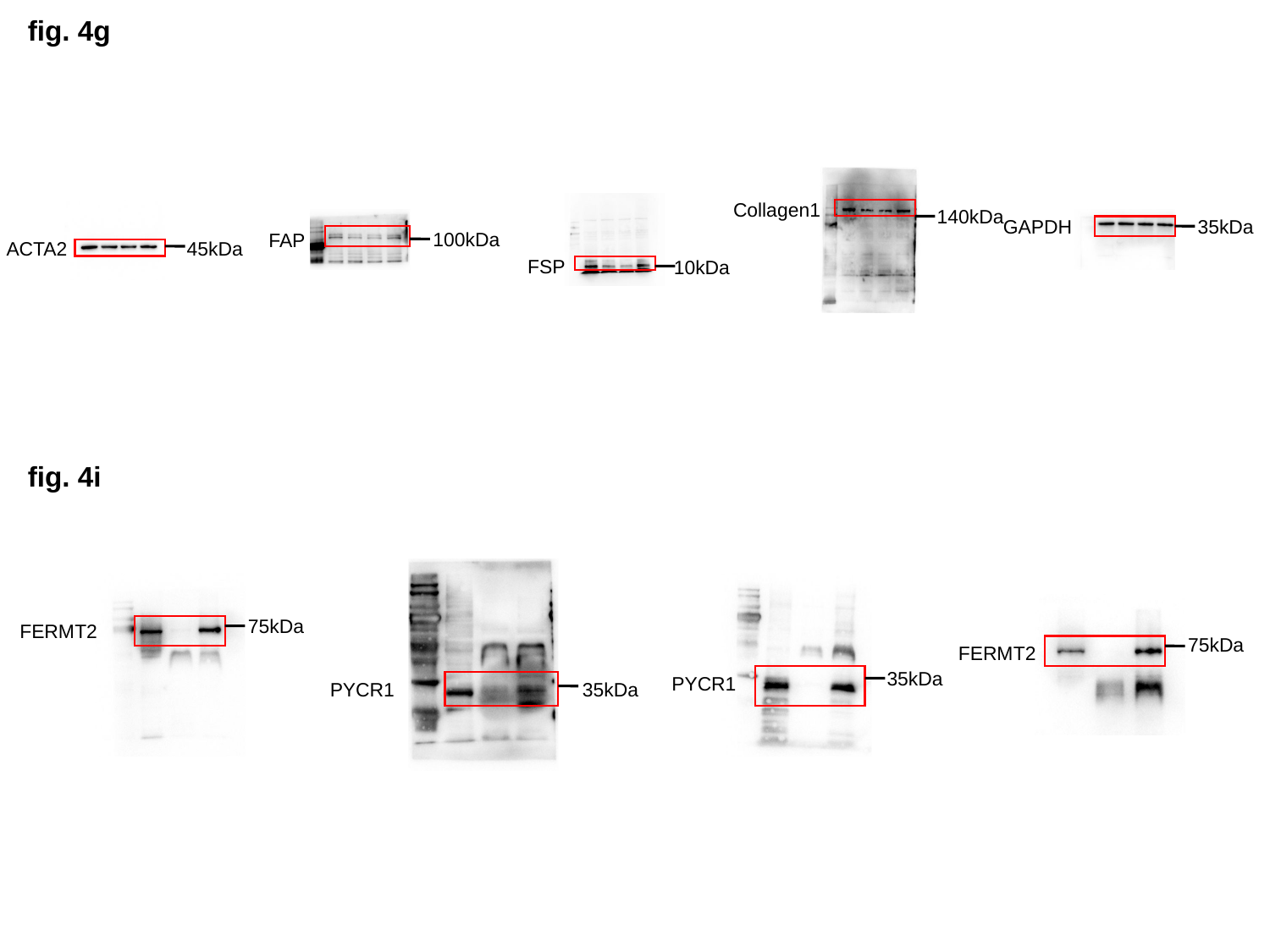

fig. 4g
Collagen1
140kDa
35kDa
GAPDH
100kDa
FAP
ACTA2
45kDa
FSP
10kDa
fig. 4i
75kDa
FERMT2
75kDa
FERMT2
35kDa
PYCR1
PYCR1
35kDa

## Slide 7
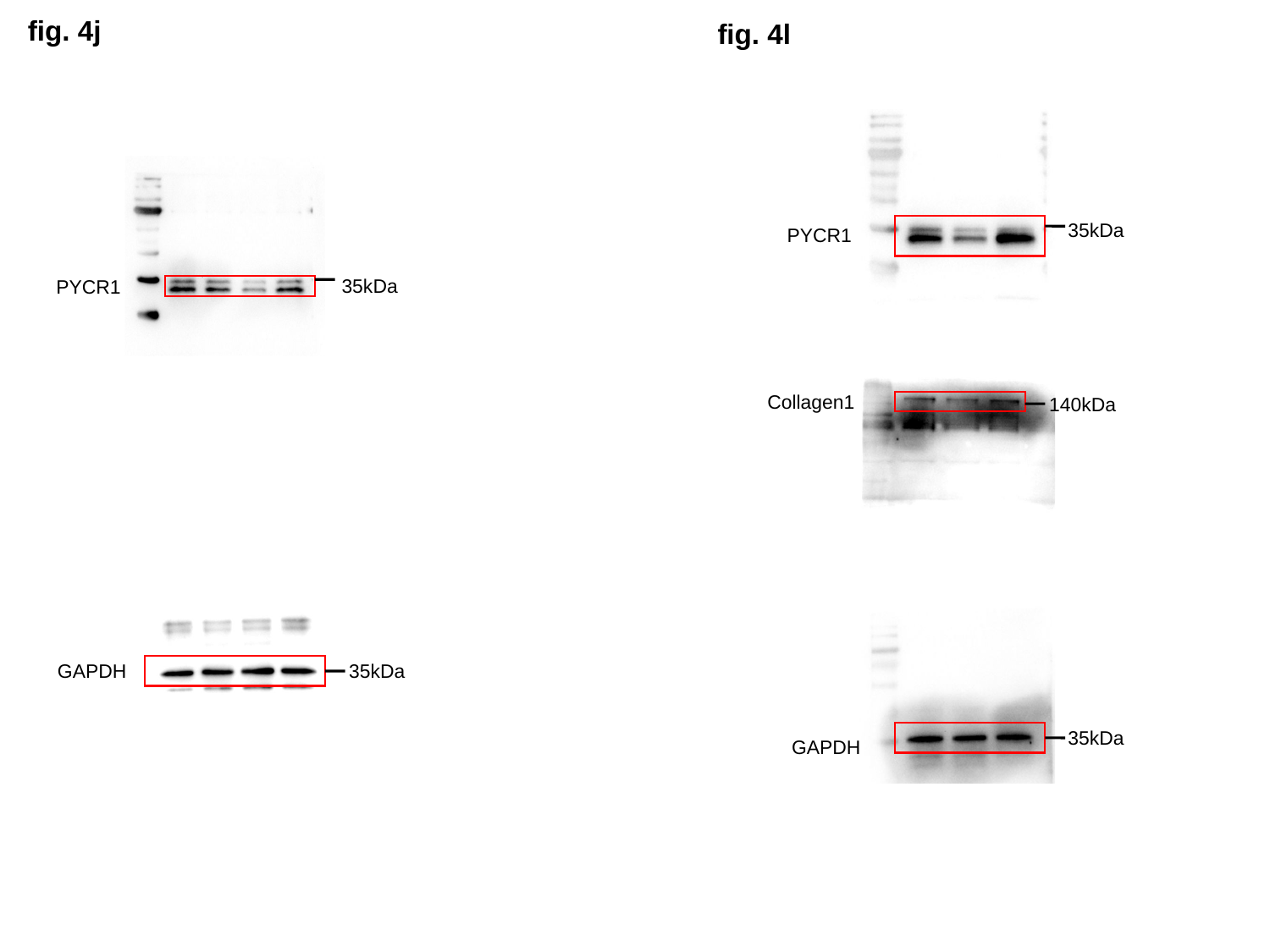

fig. 4j
fig. 4l
35kDa
PYCR1
35kDa
PYCR1
Collagen1
140kDa
GAPDH
35kDa
35kDa
GAPDH

## Slide 8
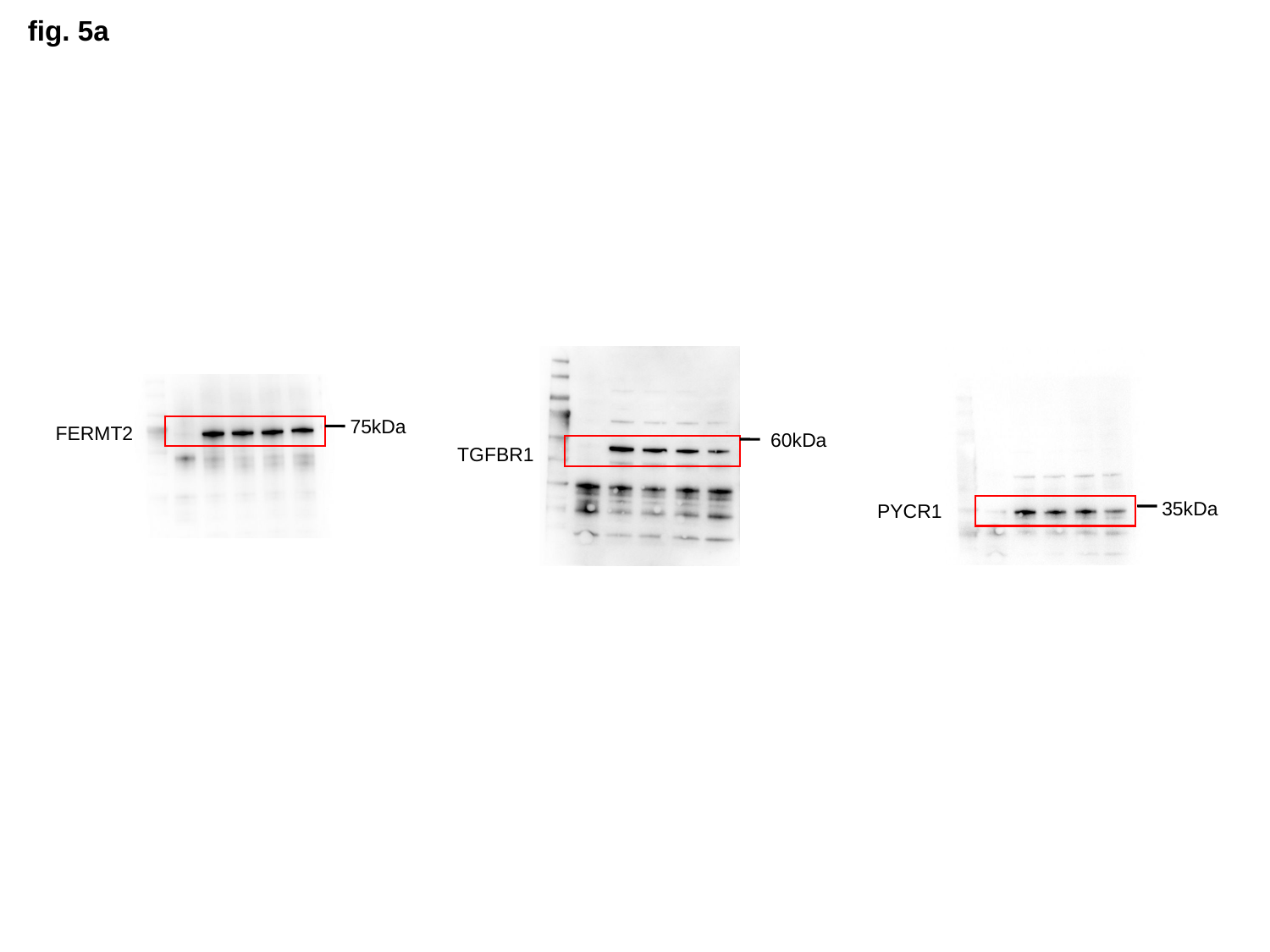

fig. 5a
75kDa
FERMT2
60kDa
TGFBR1
35kDa
PYCR1

## Slide 9
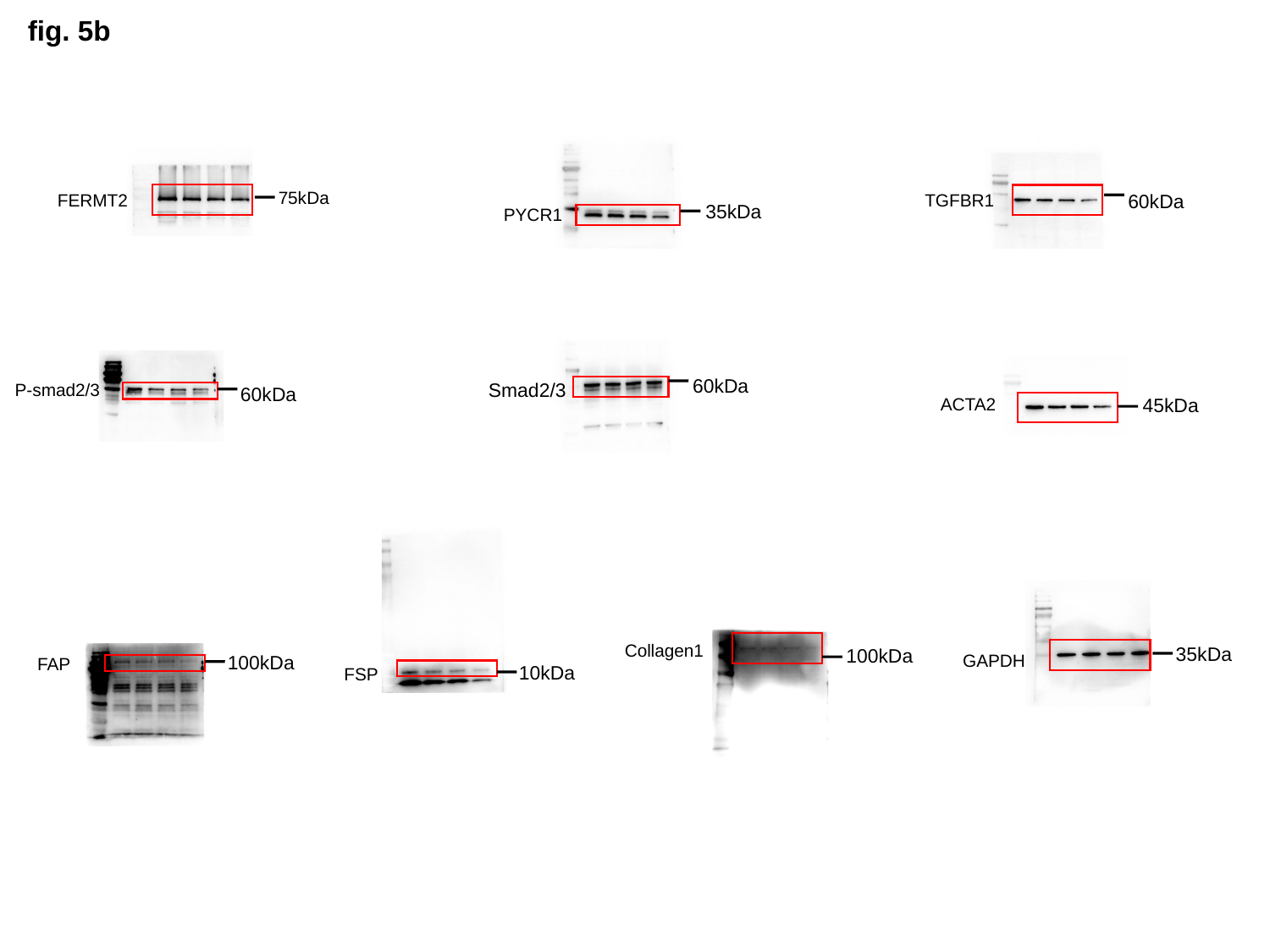

fig. 5b
75kDa
FERMT2
TGFBR1
60kDa
35kDa
PYCR1
60kDa
Smad2/3
P-smad2/3
60kDa
ACTA2
45kDa
10kDa
FSP
35kDa
GAPDH
Collagen1
100kDa
100kDa
FAP

## Slide 10
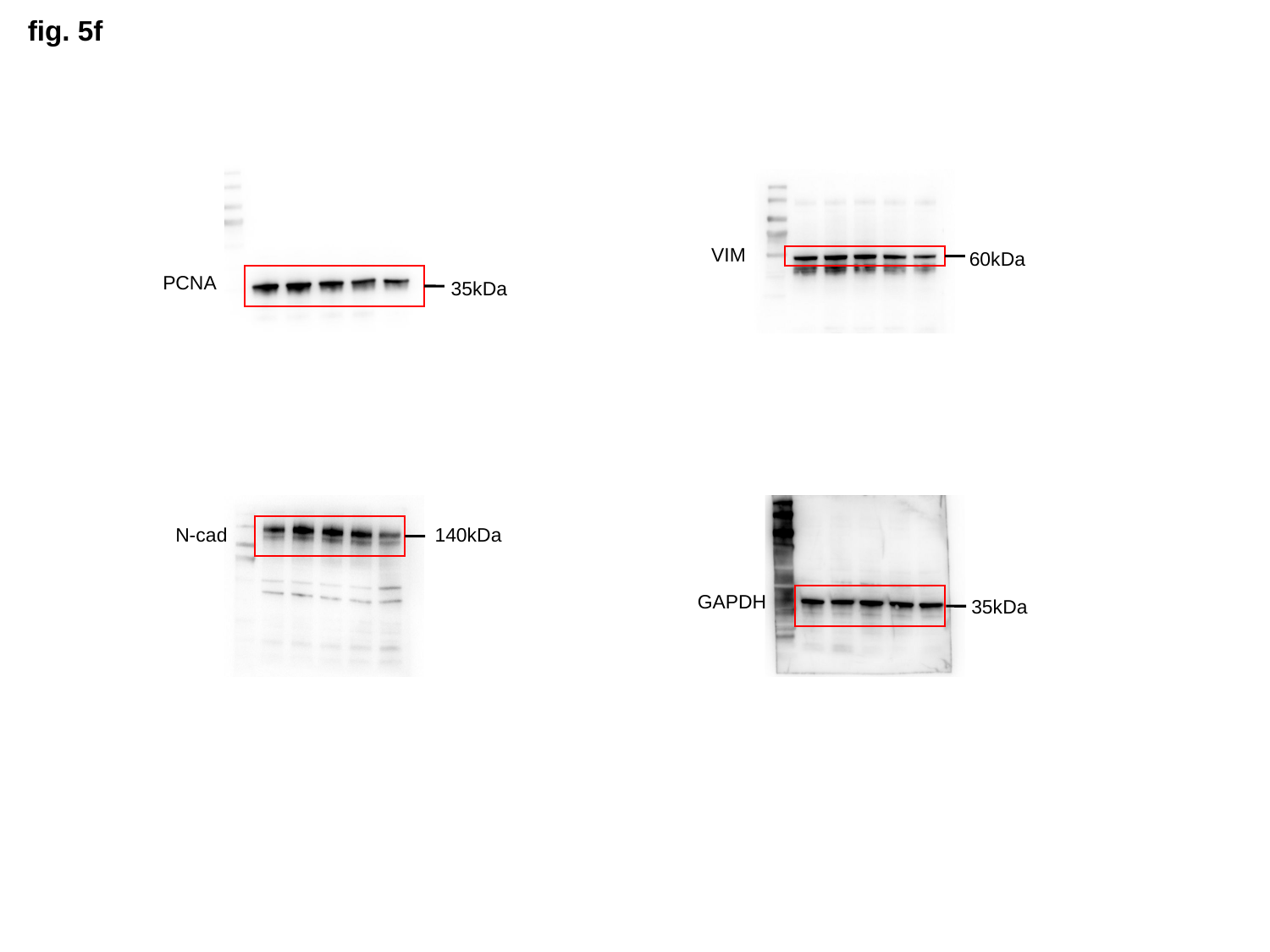

fig. 5f
VIM
60kDa
PCNA
35kDa
N-cad
140kDa
GAPDH
35kDa

## Slide 11
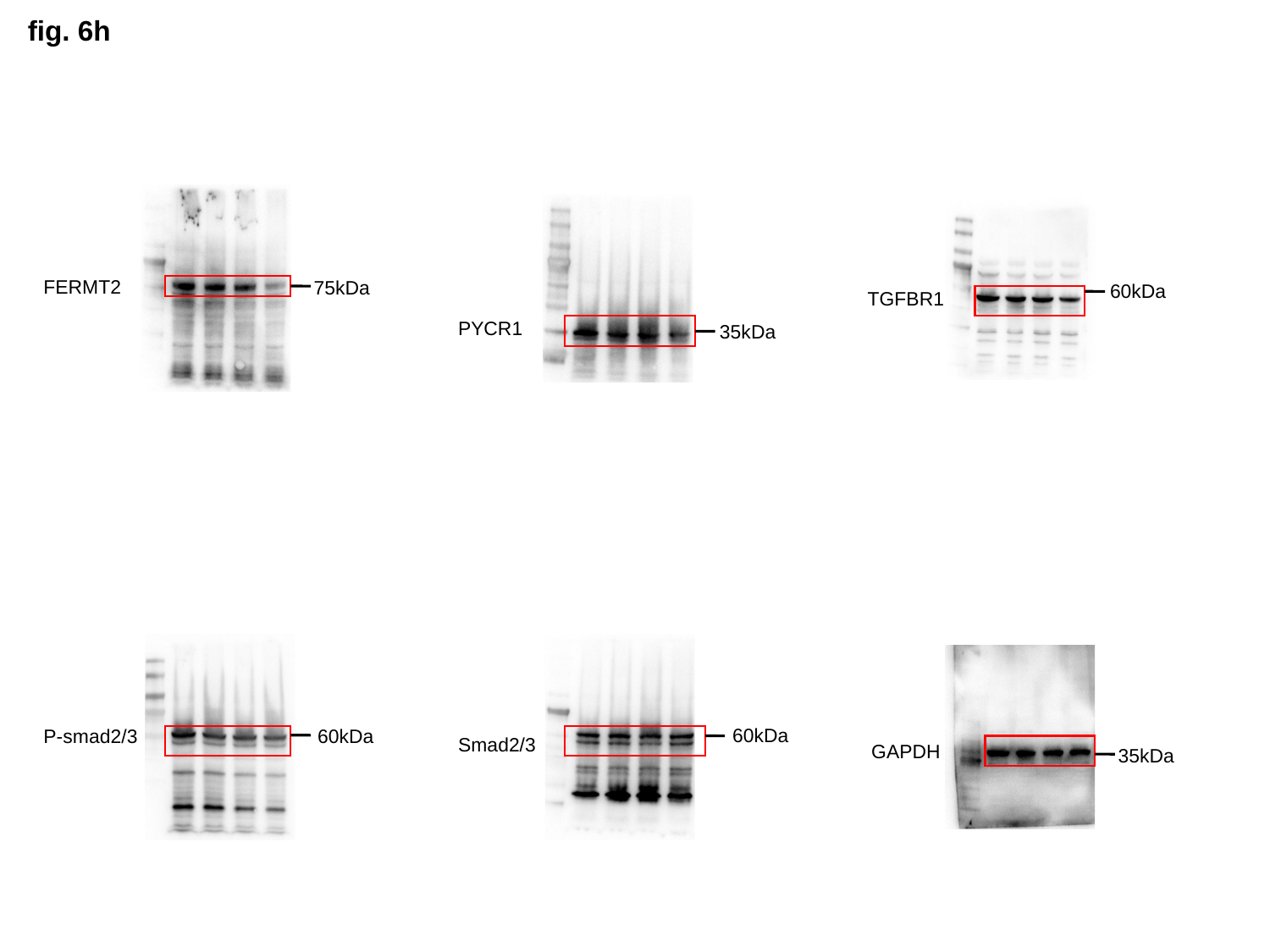

fig. 6h
FERMT2
75kDa
60kDa
TGFBR1
PYCR1
35kDa
60kDa
P-smad2/3
60kDa
Smad2/3
GAPDH
35kDa

## Slide 12
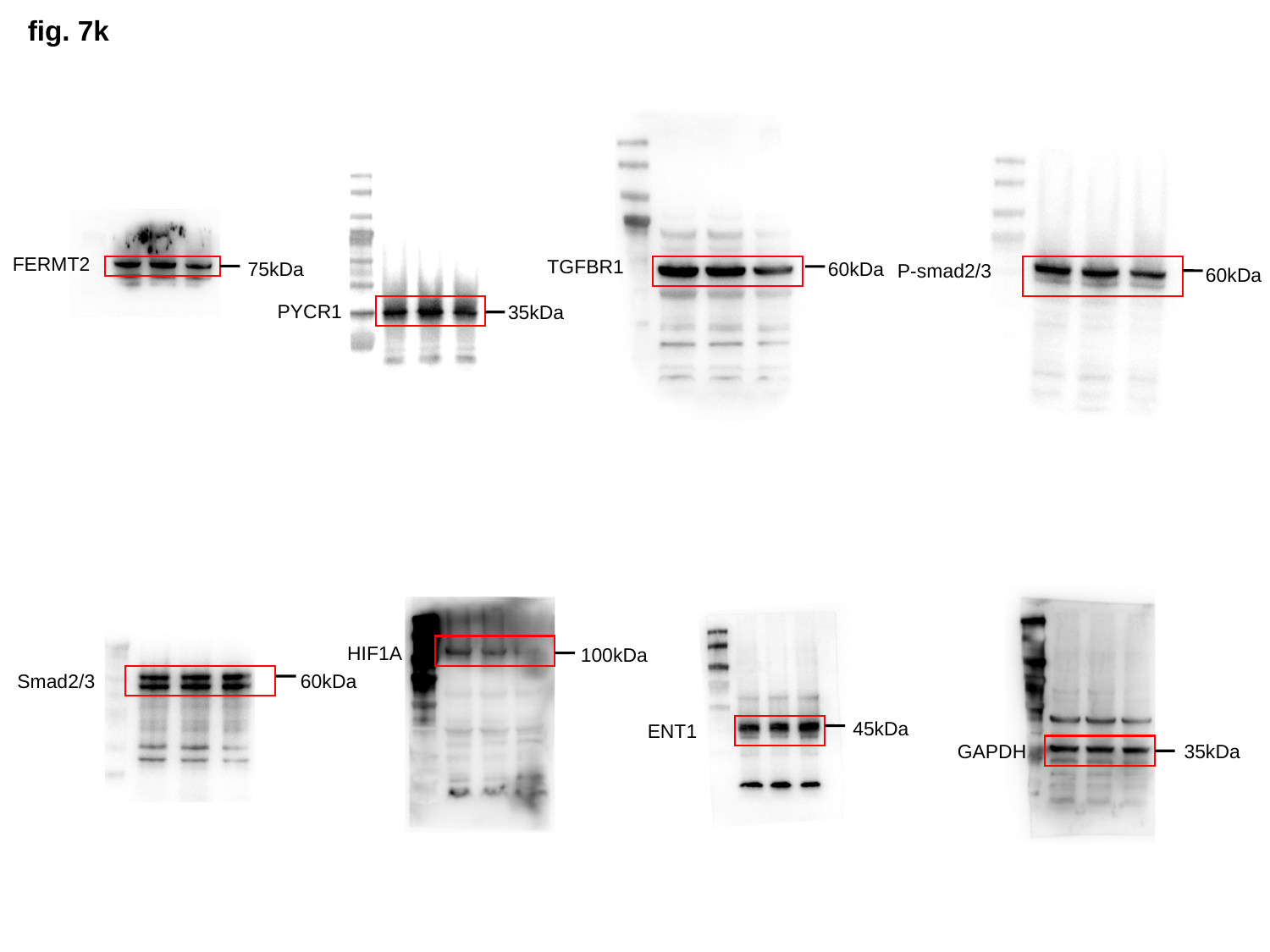

fig. 7k
FERMT2
TGFBR1
75kDa
60kDa
P-smad2/3
60kDa
PYCR1
35kDa
HIF1A
100kDa
Smad2/3
60kDa
45kDa
ENT1
GAPDH
35kDa

## Slide 13
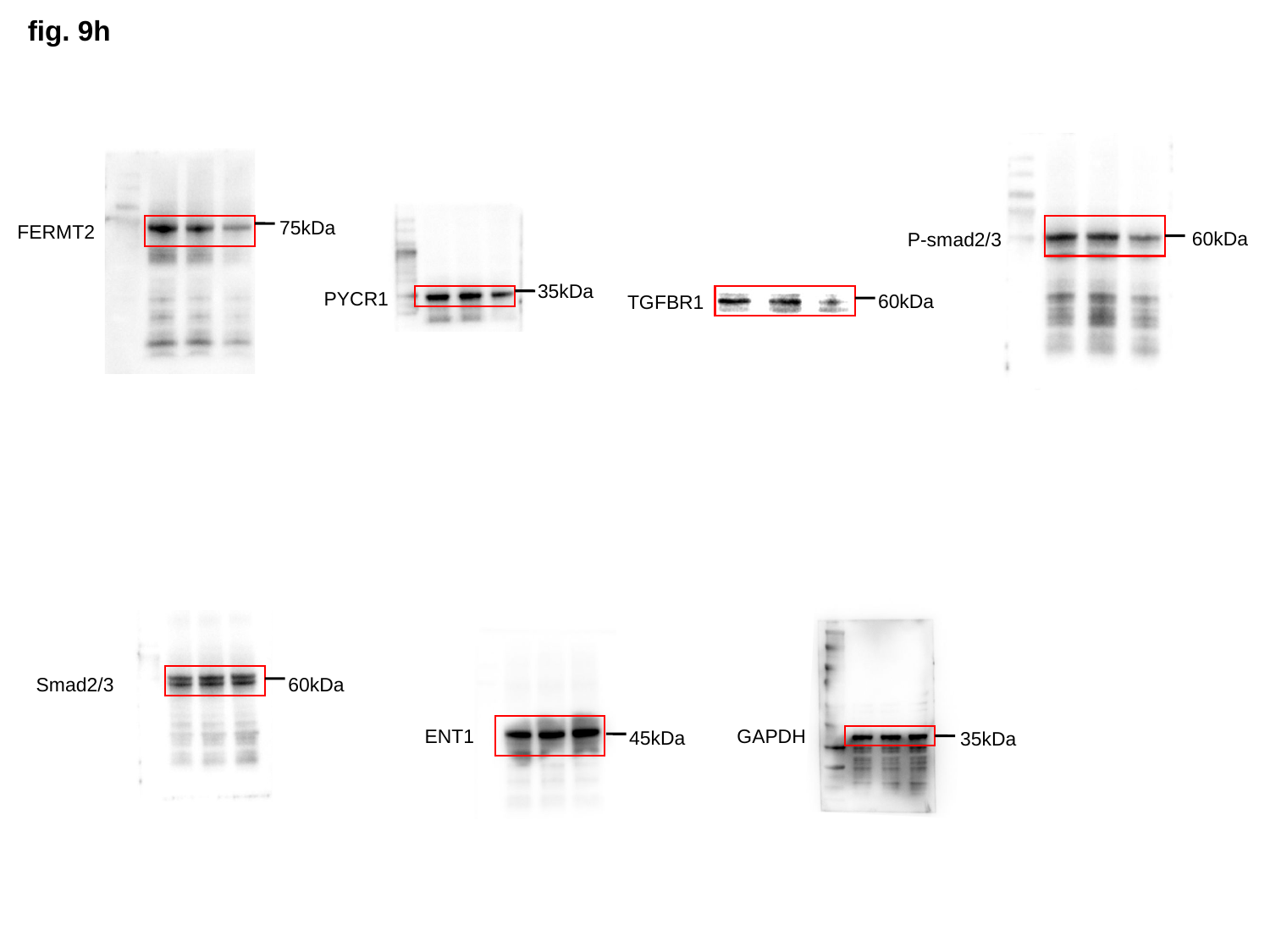

fig. 9h
75kDa
FERMT2
60kDa
P-smad2/3
35kDa
PYCR1
60kDa
TGFBR1
Smad2/3
60kDa
GAPDH
ENT1
45kDa
35kDa

## Slide 14
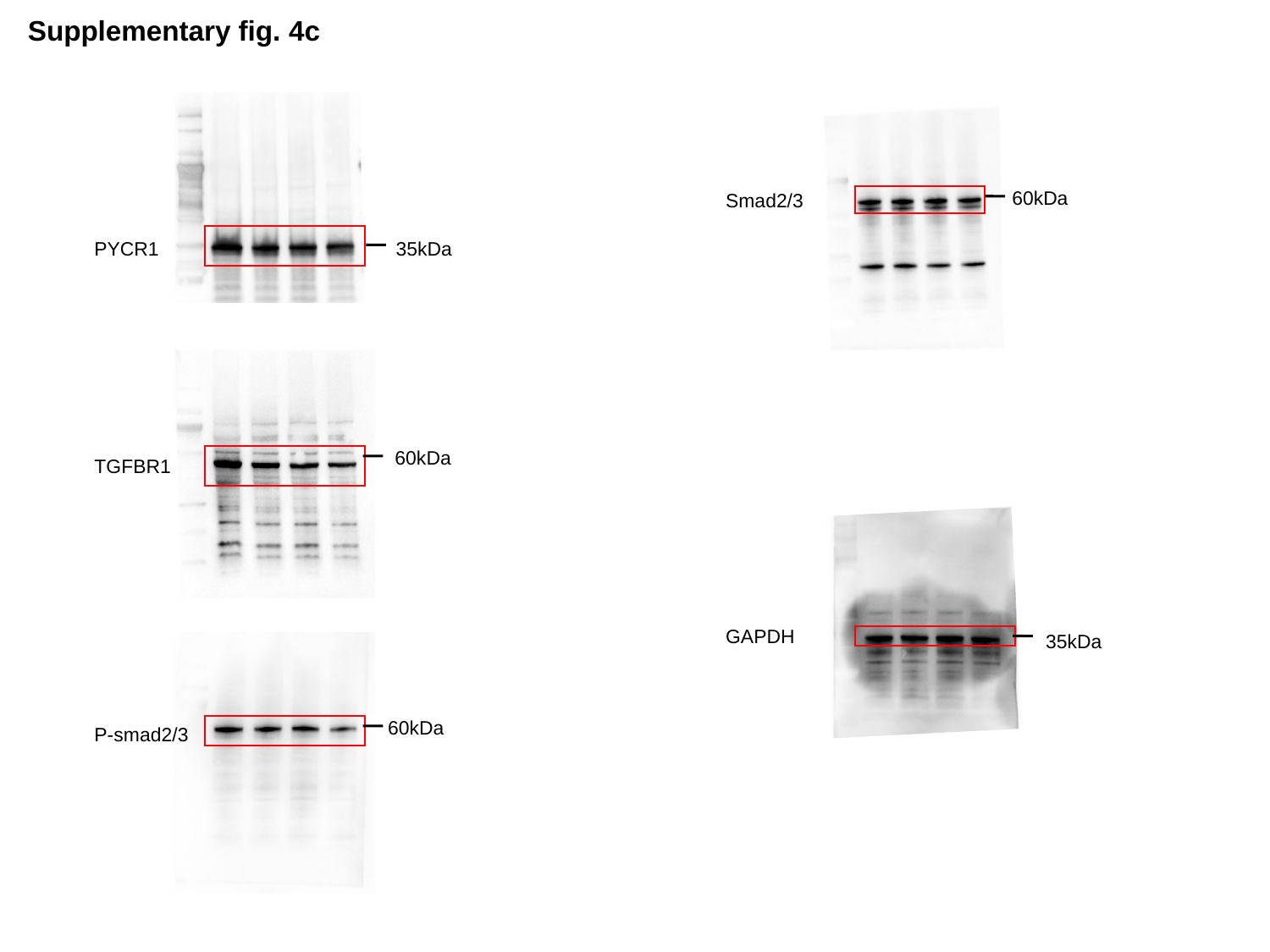

Supplementary fig. 4c
60kDa
Smad2/3
PYCR1
35kDa
60kDa
TGFBR1
GAPDH
35kDa
60kDa
P-smad2/3
